# A divide-and-conquer approach to uncover the genomic structure of the highly virulent RA strain of *Trypanosoma cruzi*

**DOI:** 10.1038/s41598-025-23742-0

**Published:** 2025-11-14

**Authors:** Virginia Balouz, Aldana Alexandra Cepeda Dean, Guadalupe Romer, Carlos Robello, Luisa Berná, Carlos Andrés Buscaglia

**Affiliations:** 1 Instituto de Investigaciones Biotecnológicas (IIBio), Universidad Nacional de San Martín (UNSAM), and Consejo Nacional de Investigaciones Científicas y Técnicas (CONICET), Av. 25 de Mayo y Francia, Campus UNSAM, B1650HMP San Martín, Buenos Aires Argentina; 2 Escuela de Bio y Nanotecnologías (EByN), UNSAM, San Martín, Buenos Aires Argentina; 3 Laboratorio de Interacciones Hospedero-Patógeno, Unidad de Biología Molecular, Institut Pasteur de Montevideo, Montevideo, Uruguay; 4 Unidad Académica de Bioquímica, Facultad de Medicina, Universidad de la República, Montevideo, Uruguay; 5 Sección de Biomatemática-Unidad de Genómica Evolutiva, Facultad de Ciencias, Universidad de la República, Montevideo, Uruguay

**Keywords:** *Trypanosoma cruzi*, RA strain, PacBio RSII, Genome assembly, Multigene family, Genome architecture, Next-generation sequencing, Parasitic infection

## Abstract

*Trypanosoma cruzi,* the causative agent of Chagas disease, remains a major health and socioeconomic concern in Latin America. Despite its remarkable genetic diversity, high-quality genome assemblies are still limited, and even widely used laboratory strains remain uncharacterized. Here, we present a high-quality genome assembly of the highly virulent RA strain (TcVI), generated using PacBio RSII long-read sequencing. Through the integration of exhaustively curated protein databases and custom-built bioinformatic tools, we improved gene annotation and achieved a comprehensive characterization of the genome. Within the RA genome we identified 17,037 genes conserved across the trypanosomatid clade and 6897 genes and pseudogenes belonging to *T. cruzi*-specific, rapidly evolving multigene families associated with host adaptation and pathogenicity. Leveraging our recently developed tool for high-throughput GC content profiling, we revealed that the RA genome is organized into 1331 isochore-like regions. This allowed, for the first time, a precise delineation of the so-called “core” and “disruptive” genomic compartments, refining previously proposed models through the identification of their exact genomic coordinates. Regions with GC content < 51%, representing approximately 45% of the genome and enriched in conserved, single-copy genes, were classified as “core”. In contrast, GC-rich regions (≥ 51%), enriched in multigene families and transposable elements, were defined as “disruptive”. Furthermore, our analysis revealed that the disruptive compartment is not homogeneous: we identified four distinct subtypes within this compartment, each characterized by specific genomic distributions, sequence compositions, and likely distinct evolutionary trajectories. This level of resolution uncovers an additional layer of genome organization complexity previously unrecognized in *T. cruzi*. The complete and deeply annotated RA strain genome thus provides a valuable resource for the research community and offers new insights into the genome architecture and evolutionary dynamics of this neglected parasite.

**Supplementary Information:**

The online version contains supplementary material available at 10.1038/s41598-025-23742-0.

## Introduction

Chagas disease, caused by the protozoan parasite *Trypanosoma cruzi*, is a vector-borne, neglected tropical illness endemic to Latin America^1^. With ~ 6.5 million people already infected and up to 120 million individuals at risk of infection, Chagas disease constitutes a major pressing public health and socioeconomic issue in endemic countries and one of the most important parasitic diseases globally^2^.

*T. cruzi* displays a quite complex population structure, with multiple strains showing differential eco-epidemiological features which were grouped into six evolutionary clades (named TcI to TcVI)^3^. Such extensive genetic variability stems from both the infrequent sexual reproduction strategy of this parasite and the plasticity of its genome. Indeed, studies based on flow cytometry and molecular karyotyping converged in revealing huge variations (up to ~ 50%) in the total DNA content of this parasite, with significant genomic differences between isolates and also among clones derived from the same strain^4,5^. In addition to structural variations, and despite *T. cruzi* being considered a diploid organism, several strains were shown to exhibit a high degree of aneuploidies, including monosomies, trisomies, and tetrasomies^6,7^. As reported in related trypanosomatids, i.e. *Leishmania* spp, these aneuploidies may be associated with drug resistance, gene expression regulation and rapid adaptation to changing environments^8,9^.

The first genome sequence for *T. cruzi* (CL Brener clone, TcVI) was produced using Sanger technology^10^. The resultant draft revealed a great complexity, with over 50% of the parasite genome consisting of repetitive regions that lack synteny, i.e., conservation of gene order and disposition, with those of phylogenetically related organisms such as *Trypanosoma brucei* and *Leishmania major*^10^. These regions are mainly represented by sequence repeats^11^, transposable elements^12^ and complex gene families that play pivotal roles in parasite niche adaptations and disease mechanisms^13,14^. *T. cruzi* multigene families are main targets of the host immune response and are accordingly under strong diversifying selection, as indicated by the presence of tens or hundreds of coding sequences (CDS) showing varying degrees of polymorphism, truncated variants (often referred to as pseudogenes) and chimeras. Indeed, genome sequencing efforts demonstrated that variations in the repertoire of multigenic families account for most of the inter-strain genetic differences in *T. cruzi*^4^.

As a general rule, *T. cruzi* CDS are arranged into directional clusters separated by strand-switch regions (SSR) where transcription directions either converge or diverge. Although no shared consensus motifs for transcription initiation and/or termination have been identified, clusters of functionally unrelated CDS lying in between contiguous SSRs define extensive polycistronic transcription units (PTU), which are subsequently processed by *trans*-splicing of a capped spliced leader RNA (SL-RNA) and polyadenylation to yield individual, mature mRNAs^15^. In addition to their role in orchestrating gene expression, recent studies have underscored an influence of SSRs in chromatin structure, transcription rate and DNA replication^16,17^**.**

Due to its repetitive nature, the CL Brener genome draft determined a highly fragmented assembly (4098 contigs, the vast majority of them < 100 Kb), in which chromosome number and structure could not be obtained^10^. Other strains were subsequently sequenced using Sanger, Illumina, Roche 454 or Ion Torrent approaches^7,18–24^. These methods generate a high number of short reads with low error rates, though they are limited in their ability to produce complete chromosome reconstructions. More recently, the application of next-generation sequencing (NGS) methods based on long reads, such as PacBio and Nanopore, has facilitated the scaffolding of long, contiguous sequences, hence significantly improving the resolution and assembly of the *T. cruzi* genome^20,25–31^. Particularly of the non-syntenic compartment, as they provided the full sequence of large clusters of highly related repeats and/or variants from multigene families without collapsing them, thereby allowing a better assessment of their dosage and variability^4^.

Long reads-based NGS technologies also revealed that the *T. cruzi* genome is organized in an isochore-like manner, i.e. fairly homogeneous stretches of DNA with differential average GC levels^25^. These regions are arranged in a mosaic-like pattern, where GC-poor segments containing trypanosomatid-conserved genes (termed ‘core’ compartments) alternate with non-syntenic, repetitive GC-rich regions throughout the genome. The latter were designated as ‘disruptive’ compartments and are characterised by the clustering of sequences from certain multigene families: mucins (TcMUC), *trans*-sialidases (TS), and mucin-associated surface proteins (MASP)^25^. Although there is certain controversy around the isochore theory^32^, such compositional genome compartmentalization has been demonstrated for a variety of taxa and is proposed to provide an ancient and fundamental level of genome organization^33^. At least in vertebrates, isochores have been shown to segregate in terms of gene composition, timing of replication^34^, overall 3-D structure^35,36^ and mutation rate, with GC-rich regions showing a higher density of transposable elements and a higher recombination frequency than GC-poor isochores^33^. In line with this, recent studies revealed that, in addition to presenting distinct gene composition, the disruptive and core parts of the *T. cruzi* genome also differ in their chromatin organization and expression pattern^16,17,37,38^.

The vast inter-strain genetic diversity precludes the definition of a ‘reference’ *T. cruzi* genome. Despite this, relatively few isolates have been sequenced to date, with genome data unavailable even for some model laboratory strains. This gap limits functional studies on diagnostic/vaccine candidates and prevents robust comparative and evolutionary genomics studies in this major human pathogen. Here, using PacBio RSII sequencing, we generated a high-quality de novo genome assembly of RA, a highly virulent and pantropic *T. cruzi* strain belonging to TcVI lineage, commonly used in Argentina-based laboratories^39–44^. We also compared the RA assembly with the genome assembly of the highly related TcVI strain TCC, which has been sequenced using the same technology^25^. Our results significantly improve the quality of the genome assembly and annotation available for this parasite and reveal previously overlooked aspects of its genome architecture.

## Results and discussion

### RA genome sequencing and assembly

Whole genome sequencing of epimastigote forms of the hybrid RA strain (TcVI) was performed using PacBio RSII technology. A total of 702,578 reads with a 9394.9 bp average length (range 35–66,694 bp) were assembled using HGAP4 software into 1815 contigs with 73 × mean coverage (Table 1). Of these, 1812 corresponded to the nuclear genome and summed to 91.2 Mpb with a gapless assembly (N50 of 132 Kb) and 51.8% GC content (Table 1). The remaining 3 contigs corresponded to mitochondrial DNA; 2 of them to maxicircles (RA_345 and RA_766, with 58,044 bp and 23,936 bp, respectively) and the third one to minicircle sequences (RA_1814, 992 bp). Completeness of the nuclear genome was assessed using Benchmarking Universal Single-Copy Orthologs (BUSCO), by mapping single-copy genes conserved in Trypanosomatid clade (trypanosoma_odb12). A total of 5383 out of 5397 BUSCO genes were identified and classified as complete. For 4901 of these genes, two copies, most likely alleles, were found, suggesting a high degree of resolution of parental haplotypes. Overall, the RA genome presented a 99.7% completeness, which is in line with the 99.8% obtained for the TCC strain^25^ , also affiliated to TcVI and with an overall 98% identity to the RA genome (Suppl. Table 1, Table 1).

**Table 1:** QUAST and BUSCO metrics of RA nuclear genome assembly and comparison with the TCC assembly (Berná et al.^25^).

Metric	RA	TCC
# contigs	1812	1237
Largest contig	881,497	1,305,230
Total length	91,255,624	87,058,484
GC (%)	51.8	51.72
N50	132,984	264,196
L50	158	92
# N’s per 100 Kb	0	0
Complete single copy genes	482	577
Complete duplicated genes	4901	4808
Fragmented genes	0	1
Missing genes	14	11
Completeness	99.7%	99.8%

N50: contig length such that using longer or equal length scaffolds produces half of the bases of the assembly. L50: minimum number of contigs that produce half of the bases of the assembly.

### RA strain nuclear genome annotation

The performance of currently used genome annotation protocols in *T. cruzi* is curtailed by the level of sequence fragmentation. In addition, these protocols often rely on the migration by homology transfer of spurious and erroneous annotations from other genomes, e.g. CL Brener. These common drawbacks are particularly troublesome for the annotation of multigene families, because their variants tend to collapse during genome assembly and also because a substantial fraction of their reference protein datasets is made up of partial, out-of-frame and/or inaccurately annotated sequences^45^. To overcome this limitation, we firstly performed a comprehensive manual curation of the sequences of 4 out of the 6 most expanded and complex multigene families (TcMUC, TS, MASP and GP63 metalloproteases) (Suppl File 1)^45,46^. The same methodology was followed to annotate other, less represented gene families such as Ser-, Ala- and Pro-rich proteins (SAP) and Small Mucin-like Genes (TcSMUG)^47,48^. Within the TS family, eight groups of CDS showing structural coherence (termed TS-GI to TS-GVIII) were defined as in^49^. A ninth group, termed ‘TS unspecified’ encompassed sequences that were not assigned to either group (Suppl File 1) . In the same line, sequences from the TcSMUG gene family were split into two robust groups (TcSMUGL and TcSMUGS) differing in their structure, expression pattern and function^48,50^. Sequences of the remaining 2 most expanded multigene families (Dispersed Gene Family-1 [DGF-1] and Retrotransposon hot spot proteins [RHS]), and of UDP-Gal/GlcNAc-dependent glycosyltransferases (GT) and Thr-, Ala-, Ser- and Val-rich proteins (TcTASV) were extracted from reference datasets^25,51,52^. Despite their relevance, some of these *T. cruzi* gene families, e.g. TcTASV and SAP, are not annotated in currently available genomes. Certain groups of transposable elements and non-coding RNAs (ncRNAs) were also annotated as per guidelines provided in Methods.

Following the consolidation of our annotation protocol (Suppl Fig. 1), we obtained 29,456 features in the RA nuclear genome. These summed up to 42.11 Mb, covering 46.5% of the genome, with 1186 out of 1812 contigs containing at least one annotated feature (Table 2 and Suppl Fig. 2). When only contigs > 50 Kb were examined (*n* = 386), the annotated fraction increased up to 52.4% (Table 2 and Suppl Fig. 2). Of these features, 19,946 corresponded to CDS: 17,037 to Trypanosomatid-conserved proteins (TCP), which either bore functional annotation (henceforth TCFP, *n* = 9258) or encode hypothetical proteins of unknown function (henceforth TCHP, *n* = 7779), and 2909 to full-length members of the above mentioned multigenic families (Table 2). The number of multigenic families’ sequences increased up to 6897 when truncated variants (pseudogenes) were considered (Tables 2 and 3).

**Table 2:** Feature counts in the RA strain nuclear genome and comparison with the TCC assembly (Berná et al.^25^).

Feature		Count		bp		% occupancy	
		RA	TCC	RA	TCC	RA	TCC
CDS		19,946	22,540	29,043,078	33,685,254	31.83	38.69
TCP	TCFP	9258	10,514	12,759,492	14,709,776	13.98	16.90
	TCHP	7779	9179	10,898,109	13,212,279	11.94	15.18
Multigenic families*	CDS	2909	2847	5,385,477	5,763,199	5.90	6.62
	pseudogenes	3988	3539	8,138,950	6,949,981	8.92	7.98
Transposable elements		3452	2946	4,674,055	4,369,633	5.12	5.02
ncRNAs		2,07	1,91	257,423	225,671	0.28	0.26
Total		**29,456**	**30,935**	**42,113,506**	**45,230,539**	**46,15**	**51,95**

*For further details see Table 3. CDS: coding sequence; TCP: trypanosomatid-conserved proteins; TCFP: trypanosomatid-conserved proteins with functional annotation; TCHP: trypanosomatid-conserved hypothetical proteins.

**Table 3:** Multigene families in the RA genome and comparison with the TCC assembly (Berná et al.^25^).

Gene product	CDS		Pseudogenes*		Total	
	RA	TCC	RA	TCC	RA	TCC
**TS**	595	634	1069 (64%)	919 (59%)	1664	1553
TS (unspecified)	43	56	97 (69%)	61 (52%)	140	117
TS-GI	17	17	24 (58%)	22 (56%)	41	39
TS-GII	143	156	465 (76%)	413 (72%)	608	569
TS-GIII	14	12	32 (69%)	29 (70%)	46	41
TS-GIV	42	42	40 (48%)	23 (35%)	82	65
TS-GV	205	221	204 (49%)	190 (46%)	409	411
TS-GVI	61	64	45 (42%)	37 (36%)	106	101
TS-GVII	17	18	79 (82%)	73 (80%)	96	91
TS-GVIII	53	48	83 (61%)	71 (59%)	136	119
MASP**	868 (28)	906 (28)	670 (42%)	657 (41%)	1566	1591
**TcMUC**	699	714	374 (34%)	364 (33%)	1073	1078
**RHS**	52	52	1016 (95%)	913 (94%)	1068	965
**GP63**	174	191	374 (68%)	307 (61%)	548	498
**DGF-1**	178	201	363 (67%)	279 (58%)	541	480
**TcSMUG**	109	86	94 (46%)	72 (45%)	203	158
TcSMUGL	70	34	34 (32%)	37 (52%)	104	71
TcSMUGS	39	52	60 (60%)	35 (40%)	99	87
**SAP**	37	35	28 (43%)	28 (44%)	65	63
**TASV**	65	86	N/A	N/A	65	86
GT***	104	106	N/A	N/A	104	106
Total	**2909**	**2847**	**3988**	**3539**	**6897**	**6578**

*Pseudogene proportion is indicated between parentheses. **MASP chimeric gene counts are indicated between parentheses. ***UDP-Gal or UDP-GlcNAc-dependent glycosyltransferase. Total values for each column are in bold.

As expected, TS, MASP, TcMUC, RHS, GP63 and DGF-1 were the most represented multigenic families in the nuclear RA genome, with 1644, 1566, 1073, 1068, 548 and 541 members, respectively (Table 3). TcSMUG, GT, TcTASV and SAP followed with 203, 140, 65 and 65 members, respectively. Among TS groups, TS-GII and TS-GV were the most numerous (608 and 409 sequences, respectively) whereas TS-GII and TS-GVII displayed the highest proportion of pseudogenes (~ 80%). Consistent with previous data^4^, all of the analyzed gene families comprised large amounts of pseudogenes, which added up to ~ 30–70% of their total sequences (Table 3). This was particularly remarkable for RHS, in which pseudogenes constitute ~ 95% of the total count of sequences, suggesting a faster evolution pace for this family. Overall, multigene families accounted for ~ 13.19 Mb (14.46%) of the RA nuclear genome (Table 2).

In addition to CDS, 3452 transposable elements and 2070 ncRNAs were identified (Table 2). The most abundant transposon was the non-autonomous Short Interspersed Repetitive Element (SIRE), with 2233 copies. Other transposons such as the Long Autonomous Terminal Repeat Retrotransposons (L1Tc), Vestigial Interposed Retroelement (VIPER), Short non-Autonomous Terminal Repeat Retrotransposons (NARTc) and cruzi-associated retrotransposon (CZAR) were represented by 512, 497, 186 and 24 sequences, respectively (Table 4). Among ncRNA sequences we identified 119 tRNAs, 329 rRNAs (rRNA 5S, rRNA 18S, Large subunit (LSU)-rRNA, etc.) and 150 copies of the SL-RNA. Other kinds of ncRNAs, including small nuclear RNAs (snRNAs) and small nucleolar RNAs (snoRNAS) such as H/ACA snoRNAs and C/D snoRNAs, were also annotated (Table 4). Overall, transposable elements and ncRNAs accounted for ~ 4.67 Mb (~ 5,12%) and ~ 257.4 Kb (~ 0.28%) of the RA nuclear genome, respectively (Table 2).

**Table 4:** Transposable elements and ncRNAs in the RA genome and comparison with the TCC assembly (Berná et al.^25^).

Transposon	RA	TCC
L1Tc	512	444
NARTc*	186	121
VIPER	497	479
SIRE*	2233	1877
CZAR	24	43
ncRNA		
tRNA	119	116
rRNA 5S	254	224
SL-RNA	150	232
snRNA	16	16
snoRNA	1418	1662

*Non-autonomous.

For comparison, the sequences from TCP, multigene families, transposons and ncRNAs were also assessed in the genome of TCC^25^ using our annotation protocol (Suppl Fig. 1). This analysis evidenced that the larger genome size of the RA strain compared to TCC does not correlate with an increase in feature content. In fact, the TCC genome assembly shows a higher percentage of occupancy (51.95%) compared to RA (46.15%) (Tables 1 and 2). As shown in Tables 2 and 3, multigene families in RA and TCC strains exhibited quite similar genomic landscapes, both in quantitative and qualitative terms. The most noticeable differences were i) a slight but consistent increase (~ 8–12%) in the number of TS, RHS, GP63 and DGF-1 pseudogenes in the RA genome; and ii) an increased dosage of TcSMUGL sequences in RA as compared to TCC (104 vs 71). A closer examination of the contigs harbouring TcSMUGL sequences however suggested that this discrepancy may arise from assembly differences between the strains (Suppl Fig. 3).

The dosages of most types of analyzed transposable elements were also rather conserved between RA and TCC; with apparent expansions of NARTc sequences in RA (186 vs 121 in TCC) and of CZAR elements in TCC (43 vs 24 in RA) (Table 4). This conservation extends to every analyzed ncRNA except for SL-RNA that was found to be more abundant in TCC than in RA (232 vs 150 copies; Table 4).

### RA genome organization

**Fig. 1:**
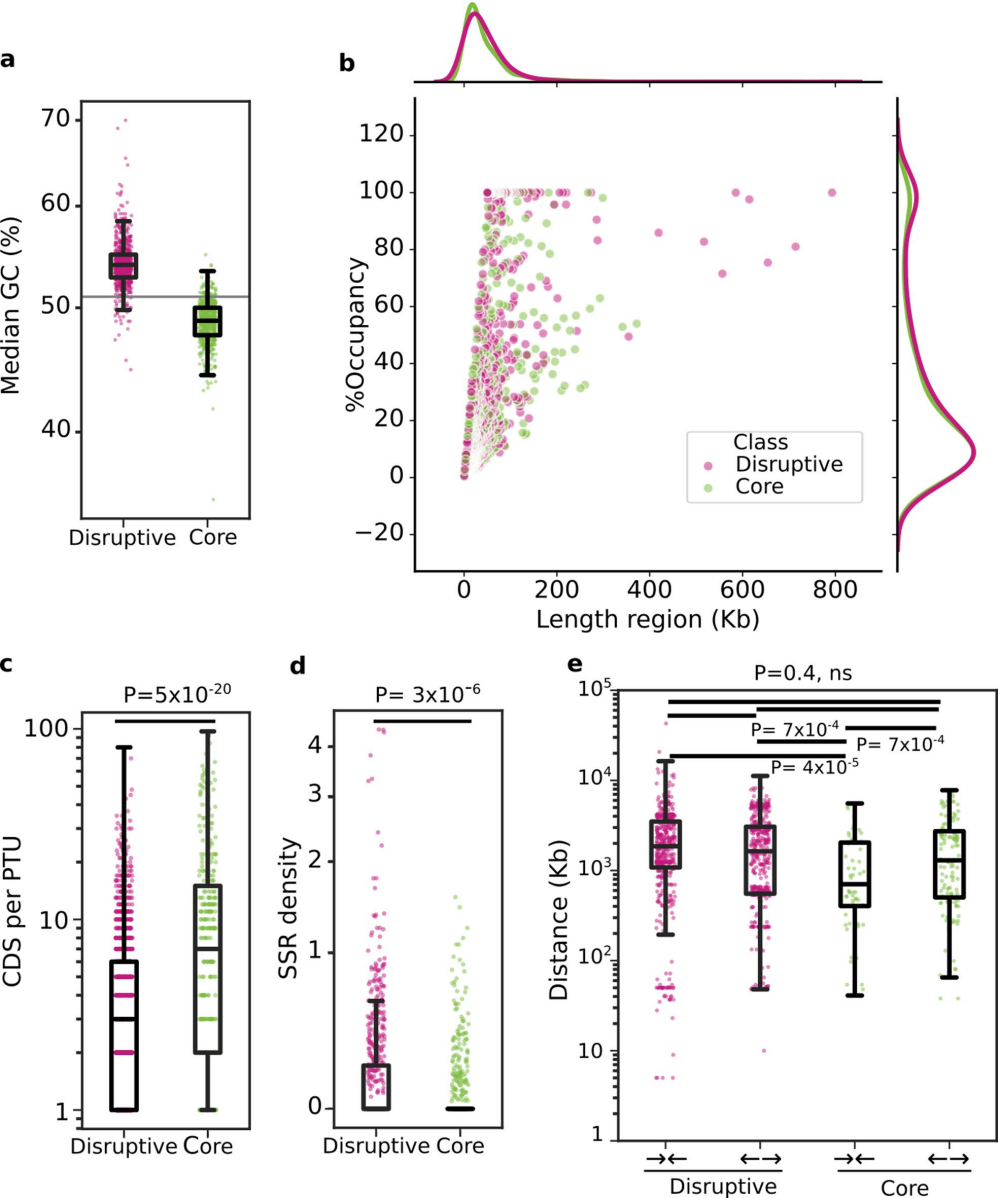
Structural features of disruptive and core regions in the RA genome. **a**, **c**, **d** and **e**. Scatter and box and whiskers plots in log scale showing the median %GC **(a)**, the CDS per PTU **(c)**, the density of SSR **(d)**, and the distance (in Kb) between genes adjacent to convergent (**C**) and divergent (**D**) SSR **(e)** in core (green) or disruptive (pink) compartments. Each box represents the first quartile, median, and third quartile, with whiskers extending 1.5 times the IQR. **b**. Jointplot displaying the percentage of occupancy of each region (calculated as its length divided by the length of the corresponding contig, multiplied by 100) according to its length. Kernel density estimate plots for disruptive (pink) and core (green) regions are shown along each axis, representing the distribution of each variable across the compartments. In** c** and **d**, *P*-values were derived from Mann–Whitney U tests comparing the indicated feature between disruptive and core regions. In **e**, Kruskal Wallis and Dunn’s post-hoc tests were performed to compare distances among compartments and SSR configurations.

To gain deeper insights into RA genome organization we used a recently developed pipeline for the high-throughput assessment of isochores on DNA datasets^53^. Using default parameters of 500 bp windows with 300 bp sliding step and a previously established 51% GC cutoff, this pipeline allows for the unbiased, i.e. independent of gene annotation, classification of core and disruptive compartments on *T. cruzi* genomes^53^. A total of 386 contigs > 50 Kb were processed, collectively spanning ~ 64.4 Mb (70.44% of the RA nuclear genome). From the 1331 regions that could be defined, 703 were classified as disruptive (smoothed GC content ≥ 0.51%) and 628 as core compartments (smoothed GC content < 51%), accounting for 55% (~ 35.5 Mb) and 45% (~ 28.9 Mb) of the analyzed genomic space, respectively (Table 5 and Fig. 1a). These figures are in the range of other *T. cruzi* genomes sequenced by long reads-based methods (Suppl Table 2). The length distribution was similar for both kinds of compartments (median length [Q1-Q3] of 31.89 [14.9–60.9] Kb for disruptive regions and 26.7 [13.9–59.6] Kb for core regions) (Table 5 and Fig. 1b). The percentage of occupancy within the corresponding contigs was also similar for core and disruptive compartments (Table 5). As previously demonstrated^25^, and at variance with *T. brucei*, disruptive regions in RA were not restricted to the subtelomeres, but distributed throughout the genome. Although most of the contigs displayed a typical mosaic structure, 63 of them were exclusively disruptive (ranging from 50.1 to 792.8 Kb), and 33 were exclusively core (ranging from 50.7 to 226.9 Kb) (Table 5). Most notably, the eight largest compartments were classified as disruptive, thus arguing for the capability of the herein used sequencing and assembly approaches in the recovery and deconvolution of repetitive regions (Fig. 1b).

**Table 5:** Disruptive and core compartments descriptive statistics.

Metric	Disruptive	Core	Significance*
n	703	628	N/A
Total bases	35,500,708	28,843,706	N/A
%GC**	54 (52.8–55)	48 (47–50)	N/A
Length (Kb)**	31.8 (14.9–60.9)	26.7 (13.9–59.6)	0.35
Contigs with such region	353	323	N/A
Contigs 100% such region	63	33	N/A
% occupancy**,***	16.8 (6.7–43.5)	15.6 (6.5–38.0)	0.1065
SSR (n)	967	189	N/A
SSR (mean)	0.18 (0.46)	0.07 (0.19)	3.7 × 10–6
Distance between genes in SSR divergent [n]**	1639.5 (553–3061) [472]	1296 (503–2726) [131]	ns
Distance between genes in SSR convergent [n]**	1856 (1856–3,95.5) [495]	714 (403.5–2042.25) [57]	3.7 × 10–4
PTUs	1226	335	N/A
CDS per PTU**	3 (1–6)	7(2–15)	5 × 10–20
Feature density (copies/10 kb)			
CDS and pseudogenes**	2.3 (1.6–3.1)	3.17 (2.3–3.8)	5.4 × 10–20
TCHP**	0.44 (0–1.1)	1.42 (0.63–1.87)	3.4 × 10–8
Transposons**	0.1 (0–0.6)	0 (0–0.3)	3.4 × 10–8
ncRNA (mean)	0.54 (3.48)	0.03 (0.23)	1.7 × 10–19

*Mann–Whitney unpaired test. **median (Q1-Q3). ***The occupancy was calculated by dividing the length of the region by the length of the contig to which it belongs, and then multiplying the result by 100. N/A: Non applicable; ns: non significant; CDS: Coding sequences; TCHP: Trypanosomatid-conserved hypothetical protein; SSR: strand-switch regions.

Taking into account the pivotal role of SSRs in *T. cruzi* DNA transcription, we assessed their distribution on the RA genome. Of note, our preliminary characterizations revealed overlaps between PTUs encoded on complementary strands of certain contigs. A closer look at these regions, however, indicated that such events most likely resulted from the migration by homology transfer of erroneously annotated sequences (Suppl Fig. 4), and the SSR defining these PTUs were thus not further considered. Global analyses indicated that core compartments present significantly less density of SSRs and more CDSs per PTU as compared to disruptive compartments (Table 5 and Fig. 1c, d). Notably, more divergent than convergent SSR were counted in core compartments (131 vs 58); a bias not observed in disruptive compartments (472 divergent SSR vs 495 convergent SSR, Table 5). The distances between CDS flanking convergent and divergent SSR in disruptive compartments, as well as those next to divergent SSR in core compartments, were relatively uniform (Table 5 and Fig. 1e). On the other hand, the distances between CDS adjacent to convergent SSR in core compartments were the shortest (Fig. 1e).

**Fig. 2:**
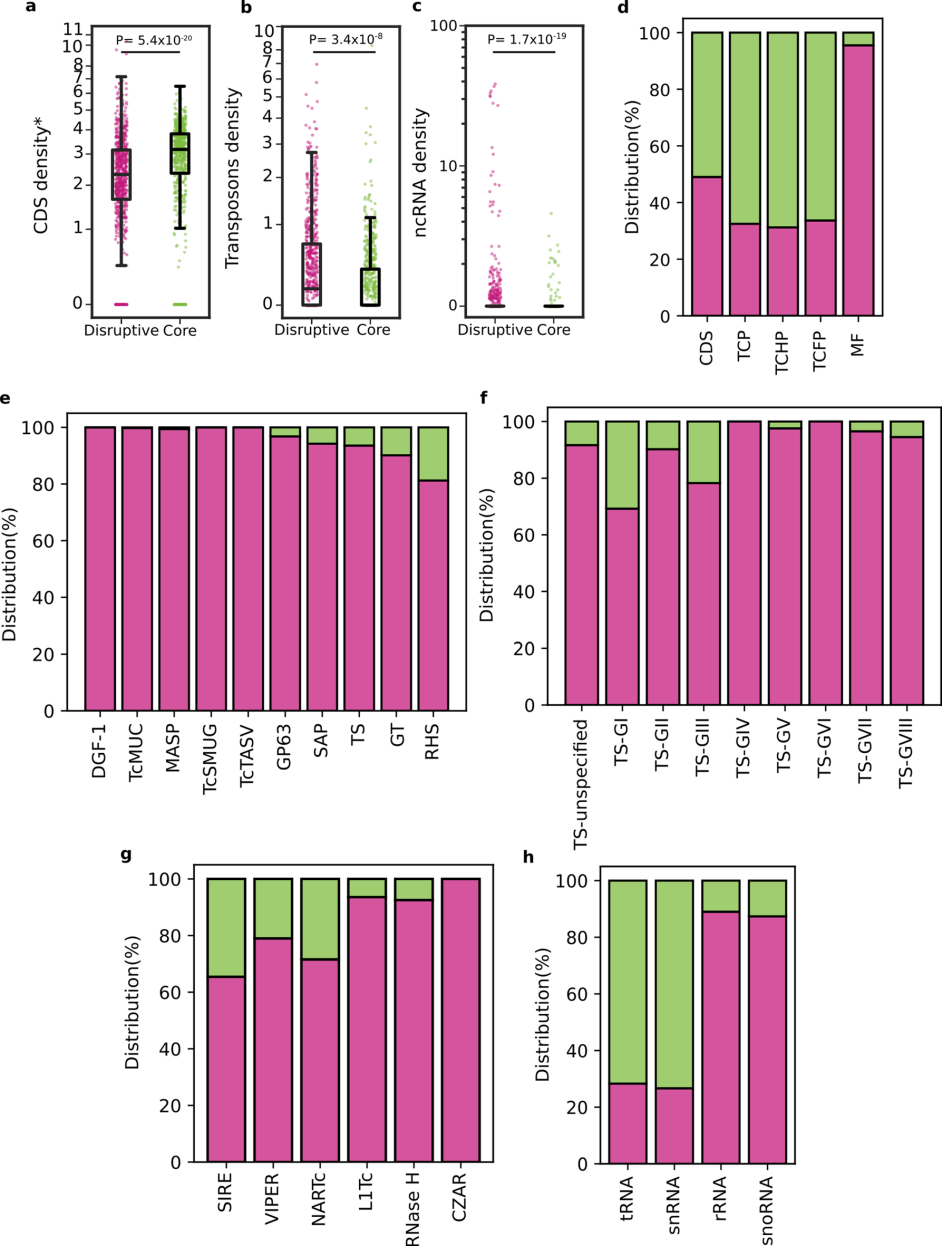
Distribution of functional features among core and disruptive regions in the RA genome. **a, b** and **c**. Scatter plots showing the density of CDS (**a**), transposon (**b**) and non-coding RNA (ncRNA) (**c**) in core (green) or disruptive (pink) compartments in log scale. Each box represents the first quartile, median, and third quartile, with whiskers extending 1.5 times the IQR for each compartment class. **d, e, f, g** and **h**. Barplots depicting the distribution as percentage of CDS and pseudogenes (**d**), multigene families (**e**), TS groups (**f**) transposon (**g**) and non-coding RNAs (ncRNAs) **(h)** in core (green) or disruptive (pink) compartments. MF: multigenic families. In **a, b** and **c**, *P*-values were derived from Mann–Whitney U tests comparing the indicated feature between disruptive and core regions. In **d** (CDS and MF data), **e** and **f**, pseudogenes are included in the analysis.

Next, we moved on to assess in more detail the effect of isochore-based compartmentalization of the RA genome on the distribution of annotated features. Though CDS were roughly equally represented in each type of compartment, GC-poor, core regions presented more density of CDS than GC-rich, disruptive regions (Table 5 and Fig. 2a). Trypanosomatid-conserved proteins (TCPs), irrespective if they have functional annotation (TCFP) or are tagged as ‘hypothetical’ (TCHP), were distributed throughout core and disruptive compartments, although they were more represented (~ 68%) in core compartments (Fig. 2d), which is consistent with the observed higher conservancy (among strains and also among trypanosomatids) of the core genome. In line with their metabolic and/or housekeeping roles, TCFP were usually found either as single-copy genes (2378; 82.56%) or as a small set of 3 to 10 highly homologous genes showing head-to-tail disposition that likely emerged by sequence duplication (441; 15%) (Suppl Table 3 and Suppl Fig. 5). It should be noted, however, that some small families of TCFP genes bearing up to 200 sequences could be identified in the RA genome (Suppl Table 4). These may be confined to a single or few contigs, i.e. histone H4 and Histone H2A, or be conspicuously distributed on the parasite genome, i.e. target of rapamycin (TOR) kinase 1 (Suppl Table 4). In addition to gene dosage and genome distribution, such small families of TCFP genes also differ in their degree of representation in core and disruptive compartments (Suppl Table 4). As a general trend, a positive correlation between gene count and accumulation in the disruptive genome may be observed (Suppl Fig. 5).

It is noteworthy the case of glycine dehydrogenase [decarboxylating] (GlyDh). On one hand, and despite the considerable size of this TCFP gene family (*n* = 61), GlyDh sequences were exclusively found in the core genome (Suppl Table 4). Moreover, inspection of GlyDh sequences revealed that they comprise solely two full-length members, showing 98.14% of identity between them, which were intriguingly non-syntenic with their orthologs in related trypanosomatids. The remaining 59 GlyDh sequences were unique to *T. cruzi* and corresponded to non-functional, fragmented variants (most likely pseudogenes), with a conspicuous core genomic distribution (Suppl Table 4). This is most striking, as it suggests i) that GlyDh amplification in *T. cruzi* was not linked to cell physiological demands, i.e. increase of protein dosage, as in other housekeeping genes; and ii) that massive amplification and pseudogenization of sequences in this organism is not a process restricted to the disruptive genome.

At variance with TCP, sequences from large multigene families were conspicuously distributed on the RA genome, though mainly in disruptive compartments (Fig. 2d). This enrichment was near absolute for DGF-1, TcMUC, MASP, TcSMUG, and TcTASV, with over 99.5% of their members located within GC-rich isochores (Fig. 2e). In this regard, it should be noted that DGF-1 genes and pseudogenes are large sequences (7–12 Kb) with a high content of GC (> 60%)^52^, which may have by themselves a major impact on local isochore definition. Other gene families such as TS, GP63, RHS, GT and SAP did not show such a strongly biased distribution, with ~ 81–97% of their sequences found in disruptive compartments (Fig. 2e and Suppl Table 3). This finding is particularly relevant in the case of TS (93.6% of association with GC-rich compartments), as it challenges the proposal of this multigenic family as a diagnostic marker of disruptive compartments^25^. When different groups of TS were analysed separately, TS-GI (30.77%), TS-GIII (21.74%), TS-GII (9.78%) and TS ‘unspecified’ (8.3%) turned out to be the most represented in core compartments (Fig. 2f and Suppl Table 3). It is particularly interesting the case of TS-GI, bearing enzymatically active TSs, in which the genomic distribution of its members seems to correlate with their evolutionary track. Briefly, the most parsimonious hypothesis states that a molecule with *trans*-sialidase activity emerged in an ancestor of the trypanosome lineage, and was readily adopted by these parasites for their interaction with arthropod vectors^54^. Following speciation, *T. cruzi* further elaborated on the TS scaffold, evolving a huge repository of polymorphic sequences (genes and pseudogenes). Even though most of them lack TS activity, they are nevertheless unified by certain structural features, including a sequence associated with tissue tropism known as FLY as well as typical bacterial/viral sialidase motifs such as Asp-boxes. Only a few TS molecules (restricted to TS-GI) retained *trans*-sialylation capacity; and a fraction of them, displaying the antigenic SAPA repeats as signature, were also repurposed as key determinants of infection and pathogenesis in the mammalian host^13,55^. Our analyses revealed that out of the 17 TS-GI CDS annotated in the RA genome, solely 11 presented the N[S/A]AYS catalytic motif and shared > 70% identity in pairwise alignments with sequences with experimentally demonstrated TS activity^56^. Of these, those likely corresponding to ‘ancestral’, insect-dwelling stages expressed variants^57^ were found in core compartments whereas those ‘novel’, i.e. bearing SAPA repeats, segregated to disruptive compartments (Suppl Table 5).

As described, transposable elements were also more represented in disruptive compartments (Fig. 2b). SIRE, VIPER, L1Tc and NARTc were predominantly (but not exclusively) found in disruptive compartments (Fig. 2g). RNaseH, which is encoded within L1Tc as part of its own retrotransposition machinery^12^, displayed the same distribution profile (Fig. 2g). In contrast, CZAR transposons were exclusively found in disruptive compartments (Fig. 2g and Suppl Table 3). As for ncRNAs, we found that tRNAs and snRNAs were predominantly found in core compartments while rRNAs and snoRNAs were mostly found in disruptive ones (Fig. 2c, h and Suppl Table 3).

**Fig. 3:**
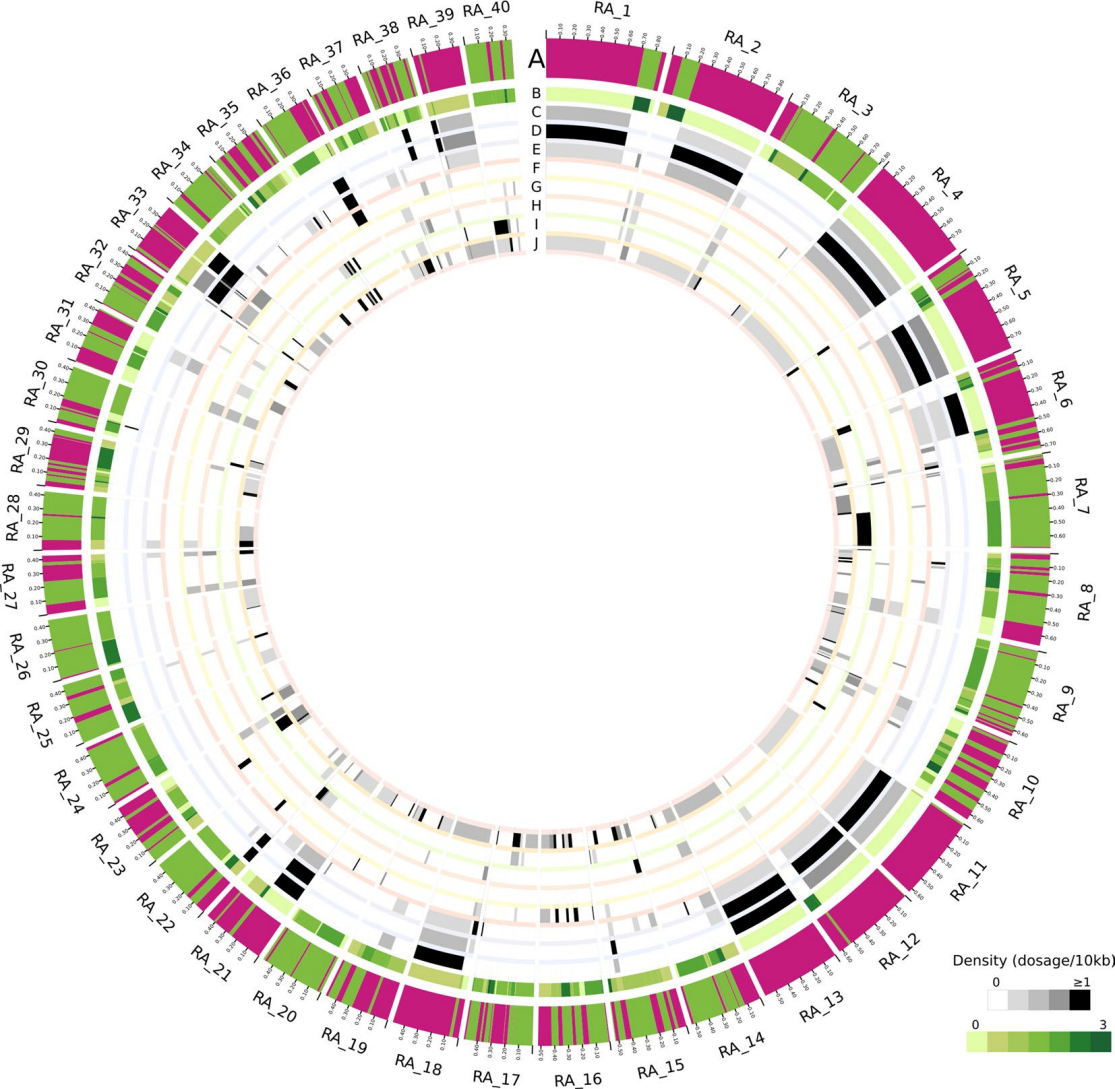
RA genome organization. **a**. In-scale circos plot of the 40 largest contigs from the RA strain. Pink and green bands indicate the disruptive (GC content > 51%) and core (GC content < 51%) genome compartments, respectively, predicted with GCanner (**A**). Densities of specific gene families and genetic elements are displayed as heatmaps: TCHP (**B**), TcMUC (**C**), MASP (**D**), TS (**E**), GP63 (**F**), DGF-1 (**G**), RHS (**H**), ncRNAs (**I**), transposons (**J**). Green and gray scales indicate the density of each feature.

Integrated data on the features (annotations of selected CDS, transposons and ncRNAs) encompassed in the 40 largest RA contigs along with the GC content-based classification for each region is shown in Fig. 3.

### Disruptive genome architecture

**Fig. 4:**
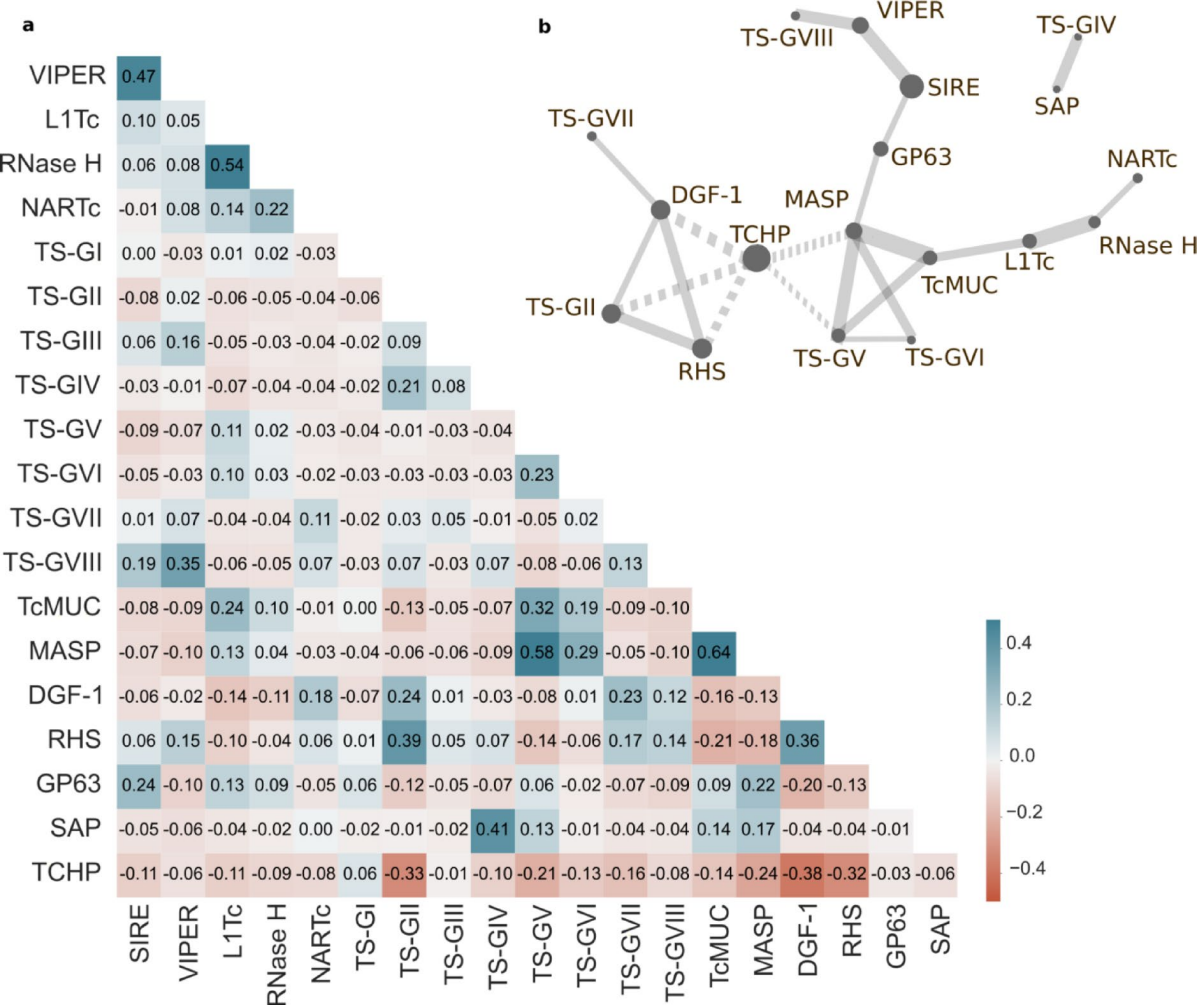
Protein association in disruptive compartments. (**a**). Correlation matrix generated using the densities of the indicated features within disruptive compartments > 20 Kb (*n* = 454), with at least one annotation. The divergent scale displays positive correlations in blue and negative correlations in red, with correlation coefficients indicated within each cell. (**b**). Robust associations (Pearson correlation > 0.21) between the genome features analyzed in **a** were summarized using a network plot. The thickness represents the absolute value of correlation and solid and dotted links depict positive and negative correlations, respectively. The dot size represents the count of disruptive regions harboring the corresponding feature.

Visual analysis of disruptive compartments revealed recurrent patterns of features’ distributions, thereby indicating putative genomic co-occurrences within regions (Fig. 3). Some of these genomic associations already have been reported, such as between MASP and TcMUC sequences^10^. To explore this issue at a genome-wide scale, we first conducted an analysis of the representation of selected features across disruptive compartments (Fig. 4a, Suppl Table 6). Correlation matrices built upon all the features’ densities in compartments > 20 Kb (*n* = 454), not only confirmed the strong TcMUC-MASP genomic association (Pearson coefficient = 0.65) but also revealed genomic co-occurrences involving other gene families/transposons. As shown in the network plot, 2 major clusters of positively correlated features could be outlined (Fig. 4b). The first one involved MASP, TcMUC, TS-GV and TS-GVI, with loose connections with GP63-SIRE-VIPER-TS-GVIII (via MASP) and L1Tc-RNaseH-NARTc, via TcMUC. Genomic association between MASP and TS-GV is of particular interest, as we have recently shown that the majority of MASP-TS chimeric genes involve TS-GV sequences^45^. Considering that TcMUC-MASP chimeras have also been demonstrated^45^, it could be hypothesized that genomic associations favor recombination events between members of these genomic families.

The second cluster involved a core of robust associations between DGF-1, RHS and TS-GII, with a weak connection to TS-GVII (via DGF-1). Both MASP/TcMUC/TS-GV/TS-GVI and DGF-1/RHS/TS-GII clusters displayed negative correlation with TCHP, which was more marked for the latter (Fig. 4b). An additional positive association was verified for TS-GIV and SAP (Pearson coefficient = 0.41), which could not be linked to any of the above mentioned clusters (Fig. 4).

**Fig. 5:**
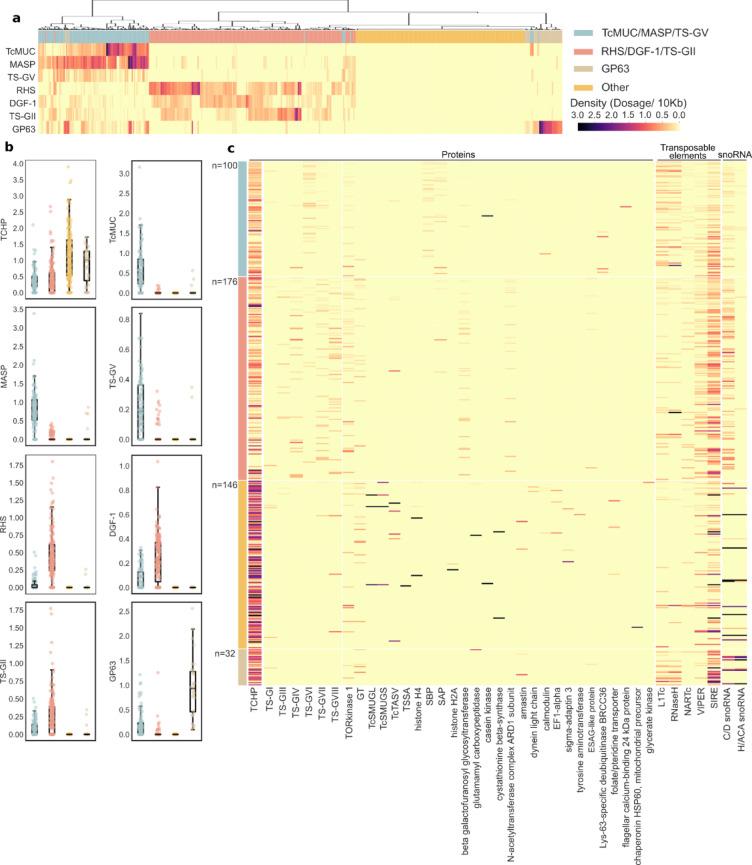
Enrichment of *T. cruzi* gene families within the genomic compartments. (**a**) Clustergram and heatmap depicting all the disruptive compartments > 20 Kb with at least one annotated feature (*n* = 454). Each column represents a region tagged as the corresponding category according to the enrichment shown (see reference). (**b**) Scatter and box and whiskers plots showing the density of TCHP, TcMUC, MASP, TS-GV, RHS, DGF-1, TS-GII and GP63 in the disruptive subcompartments. Each box represents the first quartile, median, and third quartile, with whiskers extending 1.5 times the IQR for each compartment class. (**c**). Heatmap depicting the densities of proteins, transposons and snoRNAs for each region. The regions within each category are ordered by decreasing length. EF1-alpha: Elongation factor 1 alpha; ESAG: expression site-associated gene; GT: UDP-Gal or UDP-GlcNAc-dependent glycosyltransferase; SBP: Syntaxin binding protein; SAP: Ser-, Ala- and Pro-rich proteins; TOR: target of rapamycin.

Clustergrams built upon the densities of the most represented ‘classical’ multigenic families/groups (TcMUC, MASP, TS-GV, RHS, DGF-1, TS-GII and GP63), were coherent with these correlations and allowed for the robust delineation of 4 major categories of disruptive regions in the *T. cruzi* RA genome (Fig. 5a). The first category was composed of regions with high MASP, TcMUC and TS-GV sequences, and largely overlapped the first cluster derived from the correlation matrix. This category encompassed 100 compartments, including the largest ones, and was termed ‘TcMUC/MASP/TS-GV’ (Fig. 5a). TcMUC/MASP/TS-GV regions displayed negligible or null densities of RHS, TS-GII and DGF-1 and a range of densities (from null to medium) of TCHP (Fig. 5b). Some of these regions also showed a range of GP63 densities (Fig. 5a, b). The second category, composed of 176 regions, was enriched in RHS, DGF-1 and/or TS-GII, and corresponded to the second cluster derived from the correlation matrix (Fig. 5a). This category was accordingly named as ‘RHS/DGF-1/TS-GII’ and displayed both negligible or null amounts of MASP, TcMUC and TS-GV sequences and a range of densities (from null to medium) of TCHP (Fig. 5b). RHS/DGF-1/TS-GII regions were frequently found at the terminal ends of the assembled contigs (Fig. 3), which in certain cases may correspond to telomeric/sub-telomeric chromosome regions. As described^27,31,58^, DGF-1, RHS and TS-GII are indeed enriched at *T. cruzi* telomeric/sub-telomeric positions and, due to their extremely high evolution rate and recombination frequency, they were proposed to have a major role in shaping the structure, dynamics and gene expression regulation of these regions. In line with this, it is worth noting that RHS, TS-GII and DGF-1 displayed the highest proportion of pseudogenes among multigenic groups/families in the RA genome (Table 3).

The third and less represented category (termed ‘GP63’) comprised 32 regions containing GP63 sequences (Fig. 5a). These were almost devoid of any other multigene families and contained high to very high densities of TCHP sequences (Fig. 5b). Most notably, GP63 sequences found in these compartments were different from those occasionally found in ‘TcMUC/MASP/TS-GV’ regions (see above). A recent evolutionary study of GP63 in *T. cruzi* allowed for the substructuring of this gene family into multiple groups based on sequence alignments^46^. An in-depth analysis of GP63 sequences displaying co-occurrence with ‘GP63’ or ‘TcMUC/MASP/TS-GV’ regions showed that they belong to different groups, thereby indicating that in addition to structural variations, groups of GP63 genes/pseudogenes delineated in the above study also differ in their genomic distribution (our unpublished results).

Finally, a fourth category, termed ‘Other’, was built upon the 146 regions not included in previous categories (Fig. 5a). Regions tagged as ‘Other’ were unified by their null densities of sequences from major multigene families (Fig. 5b). In addition, these regions displayed as a general trend higher densities of TCHP than those lying in other categories (Fig. 5b).

To further characterize the GC-rich, ‘disruptive’ compartments in RA, we performed heatmaps to analyze the densities of less numerous gene families (including the TS groups not considered before), TCFP gene families (Suppl Table 4), transposable elements and ncRNAs across the 4 proposed categories (Fig. 5c). This analysis supported correlation data, i.e. genomic associations between TS-GVI, L1Tc, RNaseH and SIRE with ‘TcMUC/MASP/TS-GV’ category, and unveiled previously overlooked co-occurrences of these regions with a variety of TCFP gene families, including Lys-63-specific deubiquitinase BRCC36, flagellar calcium-binding 24 kDa protein and casein kinase (Fig. 5c and Suppl Table 7). Most interestingly, it revealed the enrichment in the ‘TcMUC/MASP/TS-GV’ category of SAP sequences (Fig. 5c), involved in the invasion of mammalian cells by metacyclic trypomastigotes^47^ and syntaxin binding proteins (SBP) (Fig. 5c and Suppl Table 7), involved in the docking and fusion of vesicles in other organisms^59^. Though *T. cruzi* SBP have not been characterized, vesicle dynamics is essential for the trafficking, processing, surface disposition and shedding of GPI-anchored mucins, MASP and TS molecules^60–62^. GT sequences, involved in the elaboration of complex glycans that decorate mucins and MASP molecules and determine their functional properties^63^, were shown to be underrepresented in ‘TcMUC/MASP/TS-GV’ regions (Fig. 5c and Suppl Table 7). Low levels of C/D snoRNAs were also detected in regions lying under the ‘TcMUC/MASP/TS-GV’ category (Fig. 5c and Suppl Table 7).

On the other hand, TS-III, TS-IV, TS-VII, and TS-VIII were primarily located within regions of the ‘RHS/DGF-1/TS-GII’ category (Fig. 5c and Suppl Table 7). The N-acetyltransferase complex ARD1 subunit and ESAG-like protein families showed a similar distribution. Interestingly, ESAG proteins in *T. brucei*, which are critical for antigenic variation in this parasite, are also codified in subtelomeric regions^64^. Although not exclusively, key protein-glycosylation enzymes such as GT and beta-galactofuranosyl glycosyltransferase proteins^63^ were also highly represented in ‘RHS/DGF-1/TS-GII’ regions. In contrast to ‘TcMUC/MASP/TS-GV’ regions, the ‘RHS/DGF-1/TS-GII’ category showed a reduced representation of L1Tc (and RNAseH), SIRE and VIPER, and an overrepresentation of C/D snoRNA sequences (Fig. 5c and Suppl Table 7).

The ‘GP63’ regions showed co-occurrences with the GT gene family and, to a lesser extent, with the glutamamyl carboxypeptidase and amastin families (Fig. 5c and Suppl Table 7). As evidenced in the correlation matrix (Fig. 4), SIRE elements were also overrepresented in ‘GP63’ regions (Fig. 5c and Suppl Table 7). The role of these transposons in the transcription regulation and evolution mechanisms of the GP63 gene family was recently discussed^46^. Approximately 20% of ‘GP63’ regions showed high and concomitant density increase of C/D and H/ACA snoRNAs (Fig. 5c and Suppl Table 7). A closer inspection revealed that GP63 sequences were embedded within tandem repeats, typically comprising two H/ACA snoRNAs for each C/D snoRNA and GP63 sequence. This arrangement suggests a role for such snoRNAs in the localized amplification of GP63 sequences. Associations of some groups of *T. cruzi* GP63 with snoRNAs have been recently reported^46^.

All low copy number of *T. cruzi* specific gene families such as TcSMUG, Trypomastigote Small Surface Antigen (TSSA)^65^ and TcTASV as well as most of the small TCFP families, i.e. histones, cystathionine beta-synthase, sigma-adaptin 3, glycerate kinase, elongation factor 1-alpha, dynein light chain, chaperonin HSP60 mitochondrial precursor, folate/pteridine transporter, amastin and glutamamyl carboxypeptidase, were found on ‘Other’ regions (Fig. 5c and Suppl Tables 4 and 6). These were mostly arranged in a discrete number of tandems of highly homologous genes (likely paralogues) showing head-to-tail disposition that likely emerged by sequence duplication. These small families display low diversification, with minimal pseudogenization and/or genome translocation as compared to more complex gene families such as TS or DGF-1, suggesting a different mode of evolution. In line with this, regions from the ‘Other’ category were shown to be poor in most transposable elements, e.g. L1Tc, while exhibiting varying densities of SIRE elements (Fig. 5c and Suppl Table 7). A small proportion (~ 10%) of these regions displayed a high density of snoRNAs, which were also arranged in tandem arrays (Fig. 5c). As described above for GP63 sequences, some TCFP gene families, i.e. tyrosine aminotransferases, displayed ‘array units’ made up of the specific CDS surrounded by different kinds of snoRNAs (see RA_174 and RA_73), suggesting that these ncRNAs play an underappreciated role in *T. cruzi* genome evolution. It should also be mentioned that a few of the analyzed features, i.e. TOR kinase 1, showed a non-biased distribution across our defined categories of the disruptive *T. cruzi* genome (Fig. 5c and Suppl Table 7).

**Fig. 6:**
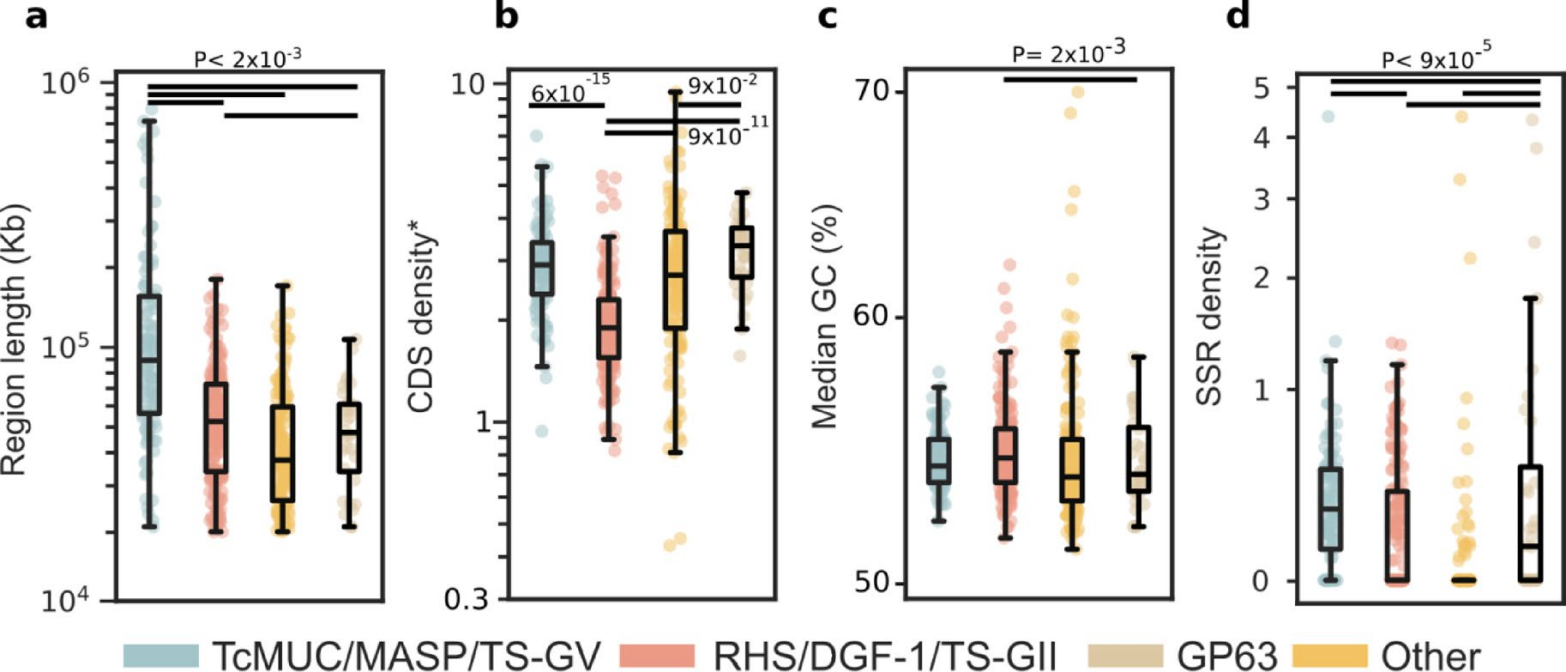
Structural features of disruptive subcompartments in the RA genome. Scatter and box and whiskers plots showing the region length (in Kb) (**a**), CDS (*and pseudogene) density (**b**), median %GC (**c**) and the density of SSR (**d**) in each kind of disruptive subcompartment (in log scale). Each box represents the first quartile, median, and third quartile, with whiskers extending 1.5 times the IQR. Statistical differences were assessed using the Kruskal–Wallis test, followed by Dunn’s post-hoc tests to compare individual features among compartments, from which the *P*-values were derived.

From a structural standpoint, the proposed categories of RA disruptive regions displayed differences in their length, CDS density, SSR density and, though to a lower extent, also in average GC content (Fig. 6). Overall, these findings, together with above shown variations in gene/feature composition and density, genomic distribution and mode of evolution, provide support to our proposed categorization of the *T. cruzi* disruptive genome.

In summary, we have generated a high-quality whole genome assembly of RA (TcVI), a virulent *T. cruzi* strain commonly used as a model in Chagas disease research laboratories. The completion and release of this genome, along with the highly curated databases underlying its annotation, will provide the scientific community with valuable resources to improve functional, epidemiological and comparative evolutionary studies into this neglected parasite. The exhaustive analysis of the RA genomic assembly, carried out using custom-built bioinformatic tools, revealed novel aspects of the *T. cruzi* genome architecture, dynamics and evolution.

## Methods

### Parasites and DNA isolation

RA epimastigotes were grown in brain heart tryptose medium supplemented with 10% fetal bovine serum at 28ºC. For DNA purification, 5 × 10^9^ epimastigotes were harvested, washed twice in 1X phosphate-saline buffer and processed according to the Quick DNA Universal kit (Zymo Research).

### Genome sequencing and assembly

PacBio library preparation and sequencing were done by the Integrative Genomics and Bioinformatics Core Beckman Research Institute, City of Hope (California, USA). Sequencing protocol was similar to the previously used for the Dm28c and TCC *T. cruzi* strains^25^. Briefly, purified DNA was mechanically fragmented using a Covaris g-TUBE device, and concentrated with AMPure PB magnetic beads. Quality assessment of the library was carried out with the Agilent 2100 Bioanalyzer device and fragments larger than 8–9 Kb were size-selected using the BluePippin device. This size threshold was shown to prevent the inclusion of prevalent kDNA minicircles (~ 20% of total *T. cruzi* DNA) in the library, thereby augmenting the sequencing depth of nuclear genomic DNA^25^. Four RSII Single Molecular Real-Time (SMRT) cells were used. The raw reads were deposited at NCBI repositories (SRA: SRR33375023, BioProject: PRJNA1256905). The de novo genome assembly was performed using the Hierarchical Genome Assembly Process version 4 (HGAP4), implemented in the SMRT Link analysis suite (Pacific Biosciences). Raw PacBio subreads were filtered and preassembled using the default seed read cutoff and length parameters. The estimated genome size was set to 110 Mb, and approximately 75 × coverage was used. The assembly step was carried out with an internal version of the Celera Assembler, and polishing was performed with the Arrow algorithm to improve consensus sequence accuracy. Quality assessment and completeness of the final assembly were performed using QUAST^66^ and BUSCO v5.7.1 (database: trypanosoma_odb12)^67^, respectively. To compare RA and TCC genome assemblies QUAST analysis were also performed. The percentage identity of alignments was calculated as the average identity across all aligned regions, extracted from the .coords file generated by QUAST. This value was computed by summing the identity scores of all alignments and dividing by the total number of alignments. The final Genome assembly file is available at the project’s GitHub public repository: github.com/BuscagliaLab/RA-genome.

### CDS annotation

Open reading frames (ORFs), defined as DNA sequences delimited by an ATG codon and a TAA, TAG, or TGA stop codon, were predicted using the GetORF tool from EMBOSS^68^. Using the settings -find 1; -minsize 120; -maxsize 21,000 we compiled all the translated ORFs into a new file that was used as input for the script 0_ORFinORF.py that scrutinized each ORF for internal Met residues defining shorter polypeptides (> 40 aa-long)^45^. Each resulting peptide was renamed, assigning a sub-index to the original ORF ID. The script also edited the coordinates to match the corresponding position of the contig. Subsequently, these sequences were mapped against a custom curated protein database (previously filtered by redundancy using the sequniq command from GenomeTools), using BLASTP^69^, setting an *E* value cutoff of 1e−10 (Suppl Fig. 1, Suppl File 1). CDS annotation was performed by assigning scores to each hit (line) in the BLASTP file (script 1_CDS-Annotation.py). Specifically, for each line on the BLASTP file a score was assigned: a score of 0 corresponded to hits whose query length matched the subject length and displayed a 100% identity. A score of 1 was assigned to a hit if the identity was > 90% and query length matched both the subject and the alignment lengths. Finally, a score of 2 was assigned to hits with > 90% identity and > 95 in both coverages defined as: i) cov1: the length of the alignment divided by the subject length and ii) cov2: the length of the alignment divided by the query length, both over 95%. All the remaining hits not fulfilling these criteria were discarded. These parameters were manually tuned and standardized to the objectives of this study. The GFF output file from script 1_CDS-Annotation.py was filtered on a second script named 2_Filtering.py. The filtering process began by selecting hits with a score of 0 and blocking the corresponding coordinates on the contig, with an added tolerance of 50 bp, to prevent CDS overlapping. After this step, hits with a score of 1 were scanned in the same order, and the coordinate-blocking process was repeated. Finally, the same procedure was applied to hits with a score of 2 (Suppl Fig. 1). Final GFF and GTF annotation files, databases and scripts are available at the project’s GitHub repository: github.com/BuscagliaLab/RA-genome.

### Pseudogene annotation

A custom curated DNA database including sequences corresponding to MASP, TS, TcMUC, TcSMUGL, TcSMUGS, DGF-1, GP63 and SAP (previously filtered by redundancy using the sequniq command from GenomeTools) was mapped against the full genome assembly using BLASTN^69^ (Suppl Fig. 1, Suppl File 1). Hits were ordered according to the alignment length using the sort command in bash and the resulting file was used as input for the 3_Pseudogene_Annotation.py script that also requires the annotated CDS (the GFF file obtained as output of the annotation script 2_Filtering.py) as input to avoid over-annotation. All the alignments covering > 20% of the subject were annotated only if bases were not occupied by a CDS. If the coverage was < 50% the script assessed if it corresponded to the N- or C-terminal portion of the molecule and annotated the result in the final GFF file. For polishing purposes, all pseudogene DNA sequences corresponding to MASP, TS, TcMUC, TcSMUGL, TcSMUGS, DGF-1, GP63 and SAP were extracted from the genome assembly, translated into protein sequence using transeq tool (EMBOSS) and manually checked to avoid including putative functional genes to the pseudogene file (Suppl Fig. 1). Sequences preserving the typical molecular signatures of the gene family, as determined by identity conservation, were manually transferred to the CDS pool (Suppl File 1).

### Transposon annotation

Retroelements belonging to CZAR, NARTc and SIRE families were annotated as described in^25^, using ad hoc generated scripts available at github.com/gaxyz/scripts-tesina. From the RepeatMasker^70^ output we selected hits corresponding to L1Tc and VIPER elements and performed a length-based filtering using Python scripts. For L1Tc elements, we conserved sequences falling within the range of 900–5,100 bp; in the case of VIPER elements, sequence annotation was restricted to those spanning from 2,000 to 6,000 bp^12^ (Suppl File 1).

### ncRNA annotation

TCC annotated sequences corresponding to tRNAs, snoRNAs and rRNAs were used as a database to perform BLASTN against the genome assembly. Using a RNA annotation Python script, information from the BLASTN output and the RNA database was compiled in a GFF file containing all annotated RNAs (Suppl File 1).

### GC content-based classification of genomic regions and feature quantification

We identified core and disruptive compartments based on GC content criteria using GCanner^53^ (https://github.com/BuscagliaLab/GC-content) and quantified the features present within each region. Briefly, GC content (expressed as percentage) was calculated in 500 bp windows with a 300 bp sliding step for each contig. These values were smoothed using locally weighted scatterplot smoothing (LOWESS) with an adjustment parameter of 100 points. After this processing, the smoothed values were classified as either above or below a cutoff threshold of 0.51, which was used to distinguish between disruptive (for values ≥ 0.51) and core compartments (for values < 0.51). This method was applied to contigs > 50 Kb, resulting in a dataframe that provided the base range (initial and final coordinates) for each compartment within each contig. The window size, step size, and cutoff threshold used to identify core and disruptive compartments were calibrated by analysing DNA sequences of varying lengths and core/disruptive ratios from the TCC strain. This calibration was performed by comparing the coordinate outputs generated by GCanner with the reference compartment schemes available at the bioinformatica.fcien.edu.uy platform. The median %GC content was calculated for each region using the output of the sliding window process (before the smoothing of data). All these data were compiled with the information available in the GFF file [function, type (CDS, RNA, transposon), strand, and start and end positions], and processed to determine descriptive values such as % occupancy (calculated by dividing the length of the region by the length of the contig to which it belongs, and then multiplying the result by 100) and feature densities (calculated as the number of events divided by the region length and normalised to 10,000 bp). For SSR counting, the strand of genes and pseudogenes embedded within each core or disruptive compartment was mapped. SSR events were classified as convergent (+ to  −) or divergent (−  to +). The last coordinate of the gene upstream and the first coordinate of the genes downstream of each SSR were used to calculate the corresponding distances. The number of genes per PTU was calculated by counting the number of consecutive + or  −  symbols. Once each region and its corresponding features were computed, the frequency of each annotated feature (e.g. TcMUC, TCHP, L1Tc, etc.) was calculated by summing its occurrences among all the core or disruptive regions (Suppl Table 3). The distribution of each feature was calculated as the percentage of events in core or disruptive compartments relative to the total number of events. For the distribution analysis of single-copy trypanosomatid-conserved functionally annotated proteins (TCFP), CDS with one or two occurrences across the genome were counted as single-copy genes. CDS present in three to ten copies were categorised as part of a small set of highly homologous genes. Their distribution across the genome was calculated as described above. Enrichment analysis was performed for each region by identifying the most densely represented multigene family of interest (TcMUC, MASP, TS-GV, RHS, DGF-1, TS-GII, and GP63).

### Data processing, visualisation, and statistical methods

All the post-annotation data processing and statistical analyses were performed using Python (v3.1). Data manipulation was carried out with Pandas (v2.2.2), and numerical operations with NumPy (v2.0.2). Data visualisations were generated using Seaborn (v0.13.2) and Matplotlib (v3.10.0) libraries. Descriptive statistics were calculated using Python’s built-in statistics module. For hypothesis testing, the Mann–Whitney U test was applied using functions from the SciPy (v1.14.1) library. When comparing more than two groups, the Kruskal–Wallis H-test from the same library was used. Post hoc comparisons were conducted using Dunn’s test with Holm correction, implemented via the scikit-posthocs package (v1.6.1). The Pearson correlation matrix (pairwise correlation) was computed using densities of the selected features across disruptive compartments larger than 20 Kb. Only disruptive regions with at least an annotated feature were included in the analysis. Hierarchical clustering of regions was performed using Seaborn’s clustermap function, with Euclidean distance as the metric and Ward’s method for linkage. Contig layouts were created using either Artemis (v17.0.1)^71^, the gggenomes R package or circos package (v0.69-9)^72^.

## Supplementary Information

Below is the link to the electronic supplementary material.

Supplementary Material 1

Supplementary Material 2

Supplementary Material 3

Supplementary Material 4

## Data Availability

Data generated and analysed during this study are included in this published article, its supplementary information files or publicly available at the project’s GitHub repository: github.com/BuscagliaLab/RA-genome/. Specifically, the following resources can be found there: Scripts: scripts.zip Annotation files: AnnotationFiles.zip Curated Databases: DBs.zip Genome Assembly: FASTA-Genomes.zip Other scripts used for the transposon annotation are available at github.com/gaxyz/scripts-tesina 25 Additionally, PacBio reads have been deposited in the Sequence Read Archive (SRA) under the accession ID SRR33375023, within the BioProject PRJNA1256905. Resources related to GCanner are available at: https://github.com/BuscagliaLab/GC-content.
