# Supplementary material for: A divide-and-conquer approach to uncover the genomic structure of the highly virulent RA strain of Trypanosoma cruzi
Source: Sci Rep. 2025 Nov 14;15:40000. doi: 10.1038/s41598-025-23742-0 (PMC12618653; doi:10.1038/s41598-025-23742-0)
Supplement: Supplementary file 2 — Supplementary Material 2 [file 41598_2025_23742_MOESM2_ESM.docx]

**Database curation**

***Trans*-sialidases**

Protein sequences of *trans*-sialidases (TS) were obtained from the curated database of the CLBrener strain available in [^1^](https://www.zotero.org/google-docs/?JeyMff). Additionally, TS sequences from TCC [^2^](https://www.zotero.org/google-docs/?PKadVC) were extracted from the annotated dataset TriTrypDB-48_TcruziTCC_AnnotatedProteins.fasta available on TritypDB, using the *‘trans*-sialidase’ as search term in the header. All sequences were compiled into a single dataset, aligned using the ClustalW online tool [^3^](https://www.zotero.org/google-docs/?HRCwhU) and visualized using Jalview (v2.11.4.1). Both the TCC and CL Brener TS datasets included sequences featuring an N-terminal addendum as described for MASP [^4^](https://www.zotero.org/google-docs/?rVsI9d). The trimming of these sequences was performed manually, based on the most closely related sequence (in terms of identity) available in the database. Following the removal of the N-terminal segment, the presence of a signal peptide (SP) was assessed using SignalP (v5.0) [^5^](https://www.zotero.org/google-docs/?fDNAo6). After editing and removing truncated sequences, the database comprised 586 and 437 sequences from TCC and CLBrener, respectively. Of these, 31 sequences corresponded to TS G-I (16 from TCC and 15 from CLBrener), 242 to TS G-II (142 from TCC and 100 from CLBrener), 22 to TS G-III (11 from each strain), 58 to TS G-IV (36 from TCC and 22 from CLBrener), 412 to TS G-V (210 from TCC and 202 from CLBrener), 98 to TS G-VI (62 from TCC and 36 from CLBrener), 32 to TS G-VII (17 from TCC and 15 from CLBrener), and 78 to TS G-VIII (42 from TCC and 36 from CLBrener). An additional 50 sequences, whose group is unspecified, were found exclusively in TCC (**Table 1,** **Supplementary File**). It is worth noting that all these sequences cluster with previously described groups (**Figure 1, Supplementary File**).

**MASP**

Protein sequences corresponding to the MASP superfamily were obtained from TCC and BrazilA4 annotated datasets [^2,6^](https://www.zotero.org/google-docs/?RHFTqO) and curated as described [^4^](https://www.zotero.org/google-docs/?wTJarI). As a result, the database comprised 878 sequences from TCC and 372 from BrazilA4 (**Table 1,** **Supplementary File**). MASP chimeras were identified during the curation of the database as described in [^4^](https://www.zotero.org/google-docs/?nv5Xbb), and manually tagged as ‘chimeric’ in the ‘gene_product’ field of the description. Nineteen chimeric sequences were identified in TCC and 11 in BrazilA4 (**Table 1,** **Supplementary File**).

**SAP**

A canonical SAP gene sequence (GenBank: DQ130019.1) [^7^](https://www.zotero.org/google-docs/?ww6aV5), was used as query in BLASTP searches against the deduced TCC proteome (generated using the 0_ORFinORF.py script) (**Supplementary Figure 1**). This search identified 32 SAP sequences in the TCC genome (**Table 1, Supplementary File**). All retrieved sequences displayed the consensus SP ‘MMCRVSFVVLVLALLCCCSCVFAV’ or closely related variants, which were bioinformatically validated using SignalP [^5^](https://www.zotero.org/google-docs/?e4CEtH). Additionally, all sequences presented the C-terminal motif ‘LHALPLLVLAALTYGTLG’, consistent with a GPI-anchor signal, as predicted by PredGPI [^8^](https://www.zotero.org/google-docs/?AeQIA5).

It is worth noting that when we used a different SAP sequence as a query (AAK30075.1, [^7^](https://www.zotero.org/google-docs/?UVIRDv)), no homologous sequences were detected in the TCC genome using the same search criteria. Further examination revealed that AAK30075.1 corresponds to an alternative reading frame within a canonical SAP sequence. Therefore, only SAP sequences derived from the DQ130019.1-based analysis were retained in the final database.


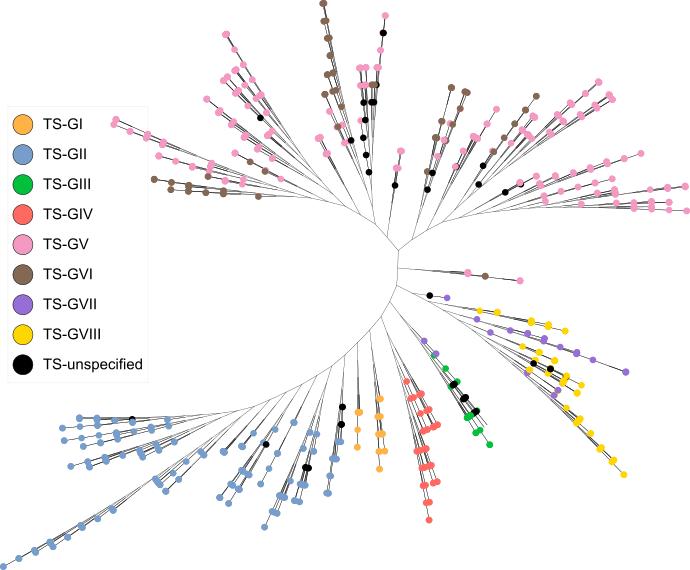


**Figure 1, Supplementary File: Unrooted gene tree of *trans*-sialidase sequences.** A total of 1,023 TS sequences from the curated protein database were aligned using the ClustalW tool [^3^](https://www.zotero.org/google-docs/?e3U56M). The resulting unrooted gene tree was visualised with iTOL [^9^](https://www.zotero.org/google-docs/?TQqYnS). Each TS sequence was colour-coded by group.

**TcSMUGL and TcSMUGS**

The curation protocol for the TcSMUG superfamily has been described [^10^](https://www.zotero.org/google-docs/?aYzhDS). Briefly, TcSMUG sequences were retrieved from the annotated protein datasets of the TCC and Dm28c strains (TriTrypDB-48_TcruziTCC_AnnotatedProteins.fasta and TriTrypDB-48_TcruziDm28c_AnnotatedProteins.fasta, respectively) by searching for the keyword “TcSMUG” in the protein headers of each FASTA file. A total of 159 sequences were aligned using ClustalW, and truncated sequences (defined as those lacking the canonical GPI-anchor signal sequence “QVPLLLLVSAAVATAGAAC” or closely related variants) were excluded. The remaining sequences were then screened for the presence of the canonical SP “MMLRRVLCVLFLALCCACVCATA”; and sequences lacking this motif (or its variants) were also removed. When an upstream peptide was found adjacent to the signal peptide, it was manually trimmed as described for TS (see above). Finally, 14 and 25 sequences from TcSMUGL and TcSMUGS groups were compiled in the protein database (**Table 1, Supplementary File**).

**GP63**

Protein sequences belonging to the GP63 superfamily were collected from the annotated datasets of the TCC strain (TriTrypDB-48_TcruziTCC_AnnotatedProteins.fasta) and AY266316.1, AY266317.1 , AY266318.1 from the CL Brener strain [^11^](https://www.zotero.org/google-docs/?WTKrgC). Sequences were aligned using ClustalW and those showing divergence from the ones reported by [^11^](https://www.zotero.org/google-docs/?KyP9KG) or lacking the consensus zinc-coordinating motif ‘HEXXH’ were removed. The final protein database comprised 170 GP63 sequences (**Table 1, Supplementary File**).

**TASV**

A subset of four CL Brener sequences, each representing one of the TASV subfamilies A, B, C, and W, was selected to perform homology searches on the RA genome (**Table 1, Supplementary File**).

**TcMUC**

TcMUCI/II curated sequences from CL Brener were obtained from [^6^](https://www.zotero.org/google-docs/?26j2js). This dataset was checked following the same approach as described for TcSMUG, searching for the canonical SP and GPI canonical signals, ‘MMTCRLLCALLVLALCCCPS’ and ‘DGSLSSSAWAFAPLVLAASALAYTAVG’, respectively [^4^](https://www.zotero.org/google-docs/?h3RCDF). The final dataset comprised 581 TcMUC curated sequences (**Table 1, Supplementary File**).

**RHS**

RHS curated sequences from CL Brener were obtained from [^12^](https://www.zotero.org/google-docs/?yOpT10). The 41 sequences were annotated according to the cluster classification proposed by the authors (**Table 1, Supplementary File**).

**DGF-1**

DGF-1 curated sequences from CL Brener were obtained from [^13^](https://www.zotero.org/google-docs/?wZqsoP).The final database comprised 127 sequences (**Table 1, Supplementary File**).

**TolT**

A subset of three CL Brener sequences, each representing one of the TolT subfamilies A, B and C [^14^](https://www.zotero.org/google-docs/?YV7075), was selected (**Table 1, Supplementary File**).

**TSSA**

A TSSAII sequence from CL Brener was included (**Table 1, Supplementary File**).

Protein sequences from above indicated multigenic families were compiled into a file, and their corresponding CDSs were retrieved by searching each protein ID in TriTrypDB. As some protein sequences had been manually trimmed during the curation process and the CDSs in the database matched the unedited versions, a custom Python script was developed to correctly match the curated protein sequences. The resulting CDS database comprised 5,294 sequences—three fewer than the protein dataset—since the three GP63 sequences reported by Cuevas et al. [^11^](https://www.zotero.org/google-docs/?kQ7d1O) were only available as protein entries in TriTrypDB.

To complete the final protein dataset with conserved Trypanosomatid proteins, additional sequences were retrieved from the TCC proteome (TriTrypDB-48_TcruziTCC_AnnotatedProteins.fasta). Sequences belonging to the multigene families mentioned above were excluded by filtering protein headers for the keywords: ‘MASP’, ‘GP63’, ‘*trans*-sialidase’, ‘TcSMUG’, ‘TcMUC’, ‘TASV’, ‘RHS’, ‘trypomastigote small surface antigen’, and ‘DGF-1’. Sequences annotated as ‘unspecified products’, corresponding to pseudogenes, were also removed. The final dataset consisted of 18,695 protein sequences and was merged with the 5,297 curated multigene family protein sequences (**Table 1, Supplementary File**).

**Table 1 Supplementary File:** Curated Database composition.

| **Gene family** | **CDS DB** | | | | **Total** | **Protein DB** | | | | **Total** |
| --- | --- | --- | --- | --- | --- | --- | --- | --- | --- | --- |
|  | **Strain** | | | |  | **Strain** | | | |  |
|  | **TCC** | **CLB** | **Brazil A4** | **Dm28c** |  | **TCC** | **CLB** | **Brazil A4** | **Dm28c** |  |
| **TS** | 586 | 437 | 0 | 0 | **1,023** | 586 | 437 | 0 | 0 | **1,023** |
| Unspecified | 50 | 0 | 0 | 0 | **50** | 50 | 0 | 0 | 0 | **50** |
| TS-GI | 16 | 15 | 0 | 0 | **62** | 16 | 15 | 0 | 0 | **62** |
| TS-GII | 142 | 100 | 0 | 0 | **484** | 142 | 100 | 0 | 0 | **484** |
| TS-GIII | 11 | 11 | 0 | 0 | **44** | 11 | 11 | 0 | 0 | **44** |
| TS-GIV | 36 | 22 | 0 | 0 | **116** | 36 | 22 | 0 | 0 | **116** |
| TS-GV | 210 | 202 | 0 | 0 | **824** | 210 | 202 | 0 | 0 | **824** |
| TS-GVI | 62 | 36 | 0 | 0 | **196** | 62 | 36 | 0 | 0 | **196** |
| TS-GVII | 17 | 15 | 0 | 0 | **64** | 17 | 15 | 0 | 0 | **64** |
| TS-GVIII | 42 | 36 | 0 | 0 | **156** | 42 | 36 | 0 | 0 | **156** |
| **MASP** | 878 | 0 | 372 | 0 | **1,250** | 878 | 0 | 372 | 0 | **1,250** |
| **MASP chimera** | 19 | 0 | 11 | 0 | **30** | 19 | 0 | 11 | 0 | **30** |
| **TcMUC** | 0 | 581 | 0 | 0 | **581** | 0 | 581 | 0 | 0 | **581** |
| **RHS** | 0 | 41 | 0 | 0 | **41** | 0 | 41 | 0 | 0 | **41** |
| **GP63** | 167 | 0 | 0 | 0 | **167** | 167 | 3 | 0 | 0 | **170** |
| **DGF-1** | 0 | 127 | 0 | 0 | **127** | 0 | 127 | 0 | 0 | **127** |
| **TcSMUGL** | 7 | 0 | 0 | 7 | **14** | 7 | 0 | 0 | 7 | **14** |
| **TcSMUGS** | 17 | 0 | 0 | 8 | **25** | 17 | 0 | 0 | 8 | **25** |
| **SAP** | 32 | 0 | 0 | 0 | **32** | 32 | 0 | 0 | 0 | **32** |
| **TASV** | 0 | 4 | 0 | 0 | **4** | 0 | 4 | 0 | 0 | **4** |
| **TolT** | 0 | 3 | 0 | 0 | **3** | 0 | 3 | 0 | 0 | **3** |
| **TSSA** | 1 | 0 | 0 | 0 | **1** | 1 | 0 | 0 | 0 | **1** |
| **Others** | 0 | 0 | 0 | 0 | **0** | 18,695 | 0 | 0 | 0 | **18,695** |
| **Total** | **1,707** | **1,193** | **383** | **15** | **3,298** | **20,402** | **1,196** | **383** | **15** | **21,996** |

* The CDS corresponding to the GP63 sequences reported in [^11^](https://www.zotero.org/google-docs/?wBaXLb) are not available in public databases.

**References**

[1. Freitas, L. M. *et al.* Genomic Analyses, Gene Expression and Antigenic Profile of the Trans-Sialidase Superfamily of Trypanosoma cruzi Reveal an Undetected Level of Complexity. *PLOS ONE* **6**, e25914 (2011).](https://www.zotero.org/google-docs/?4nNSlt)

[2. Berná, L. *et al.* Expanding an expanded genome: long-read sequencing of Trypanosoma cruzi. *Microb. Genomics* **4**, e000177 (2018).](https://www.zotero.org/google-docs/?4nNSlt)

[3. Higgins, D. G., Thompson, J. D. & Gibson, T. J. Using CLUSTAL for multiple sequence alignments. *Methods Enzymol.* **266**, 383–402 (1996).](https://www.zotero.org/google-docs/?4nNSlt)

[4. Dean, A. A. C., Berná, L., Robello, C., Buscaglia, C. A. & Balouz, V. An algorithm for annotation and classification of T. cruzi MASP sequences: towards a better understanding of the parasite genetic variability. *BMC Genomics* **26**, 194 (2025).](https://www.zotero.org/google-docs/?4nNSlt)

[5. Almagro Armenteros, J. J. *et al.* SignalP 5.0 improves signal peptide predictions using deep neural networks. *Nat. Biotechnol.* **37**, 420–423 (2019).](https://www.zotero.org/google-docs/?4nNSlt)

[6. Wang, W. *et al.* Strain-specific genome evolution in Trypanosoma cruzi, the agent of Chagas disease. *PLoS Pathog.* **17**, e1009254 (2021).](https://www.zotero.org/google-docs/?4nNSlt)

[7. Baida, R. C. P. *et al.* Molecular Characterization of Serine-, Alanine-, and Proline-Rich Proteins of Trypanosoma cruzi and Their Possible Role in Host Cell Infection. *Infect. Immun.* **74**, 1537–1546 (2006).](https://www.zotero.org/google-docs/?4nNSlt)

[8. Pierleoni, A., Martelli, P. L. & Casadio, R. PredGPI: a GPI-anchor predictor. *BMC Bioinformatics* **9**, 392 (2008).](https://www.zotero.org/google-docs/?4nNSlt)

[9. Letunic, I. & Bork, P. Interactive Tree of Life (iTOL) v6: recent updates to the phylogenetic tree display and annotation tool. *Nucleic Acids Res.* **52**, W78–W82 (2024).](https://www.zotero.org/google-docs/?4nNSlt)

[10. Bertotti, S. *et al.* Characterization of ADAT2/3 molecules in Trypanosoma cruzi and regulation of mucin gene expression by tRNA editing. *Biochem. J.* **479**, 561–580 (2022).](https://www.zotero.org/google-docs/?4nNSlt)

[11. Cuevas, I. C., Cazzulo, J. J. & Sánchez, D. O. gp63 homologues in Trypanosoma cruzi: surface antigens with metalloprotease activity and a possible role in host cell infection. *Infect. Immun.* **71**, 5739–5749 (2003).](https://www.zotero.org/google-docs/?4nNSlt)

[12. Bernardo, W. P. *et al.* Genomic Organization and Generation of Genetic Variability in the RHS (Retrotransposon Hot Spot) Protein Multigene Family in Trypanosoma cruzi. *Genes* **11**, 1085 (2020).](https://www.zotero.org/google-docs/?4nNSlt)

[13. Kawashita, S. Y., da Silva, C. V., Mortara, R. A., Burleigh, B. A. & Briones, M. R. S. Homology, paralogy and function of DGF-1, a highly dispersed Trypanosoma cruzi specific gene family and its implications for information entropy of its encoded proteins. *Mol. Biochem. Parasitol.* **165**, 19–31 (2009).](https://www.zotero.org/google-docs/?4nNSlt)

[14. Lobo, M. *et al.* Molecular and antigenic characterization of Trypanosoma cruzi TolT proteins. *PLoS Negl. Trop. Dis.* **13**, e0007245 (2019).](https://www.zotero.org/google-docs/?4nNSlt)
