# Supplementary material for: A divide-and-conquer approach to uncover the genomic structure of the highly virulent RA strain of Trypanosoma cruzi
Source: Sci Rep. 2025 Nov 14;15:40000. doi: 10.1038/s41598-025-23742-0 (PMC12618653; doi:10.1038/s41598-025-23742-0)
Supplement: Supplementary file 4 — Supplementary Material 4 [file 41598_2025_23742_MOESM4_ESM.docx]

**SUPPLEMENTARY MATERIAL**

**Supplementary Table 1. Comparison of RA and TCC genome assemblies.**

| **Assembly*** | **RA vs TCC** |
| --- | --- |
| **Length** | 91,255,624 vs 87,058,484 |
| **GC (%)** | 51,8 vs 51,72 |
| **# misassemblies** | 1523 |
| **# misassembled contigs** | 839 |
| **Misassembled contigs length** | 64,619,134 |
| **# local misassemblies** | 442 |
| **# scaffold gap ext. mis.** | 0 |
| **# scaffold gap loc. mis.** | 0 |
| **# unaligned mis. contigs** | 8 |
| **# unaligned contigs** | 5 + 226 part |
| **Unaligned length** | 1,771,090 |
| **# mismatches per 100 kbp** | 456.26 |
| **# indels per 100 kbp** | 284.93 |
| **Largest alignment** | 743,507 |
| **Total aligned length** | 86,888,904 |
| **NA50** | 66,551 |
| **NGA50** | 71,602 |
| **NA90** | 7,773 |
| **NGA90** | 11,336 |
| **auNA** | 105253.1 |
| **auNGA** | 110327.5 |
| **LA50** | 344 |
| **LGA50** | 313 |
| **LA90** | 2,017 |
| **LGA90** | 1,616 |

* Metrics were calculated following QUAST analysis using TCC as reference genome [^25^](https://www.zotero.org/google-docs/?qOIckH).

**Supplementary Table 2. Genome compartmentalization in *T. cruzi* strains.**

| **Strain** | **Core** | **Disruptive** | **Bases core (Kp)** | **Bases disruptive (Kp)** |
| --- | --- | --- | --- | --- |
| **RA** | 44.82 | 55.17 | 28,843.7 | 35,500.7 |
| **TCC*** | 42.02 | 57.98 | 30,009.6 | 41,405.86 |
| **Dm28c (2018)*** | 42.05 | 57.95 | 18,306.27 | 25,231.42 |
| **Y C6**** | 39.61 | 60.39 | 16,861.25 | 25,707.98 |
| **Brazil A4**** | 44.55 | 55.45 | 17,432.82 | 21,694.57 |
| *Berná et al. 2018 | |  |  |  |
| **Wang et al. 2021 | |  |  |  |

**Supplementary Table 3. Core and disruptive distribution of annotated features on the RA genome.**

See the Supplementary_table_3.csv file in https://github.com/BuscagliaLab/RA-genome

**Supplementary Table 4. Distribution of TCFP gene families on the RA genome.**

| **Functional annotation*** | **Core** | **Disruptive** | **Total count** | **Core (%)** | **Disruptive (%)** | **Contigs (n)** | **Ratio**** |
| --- | --- | --- | --- | --- | --- | --- | --- |
| ***protein kinase*** | 160 | 40 | 200 | 80 | 20 |  |  |
| target of rapamycin (TOR) kinase 1 | 20 | 96 | 116 | 17.24 | 82.76 | 81 | 1.43 |
| ***RNA-binding protein*** | 68 | 3 | 71 | 95.77 | 4.23 |  |  |
| ***Endonuclease-reverse transcriptase*** | 3 | 63 | 66 | 4.55 | 95.45 |  |  |
| glycine dehydrogenase [decarboxylating] | 61 | 0 | 61 | 100 | 0 | 27 | 2.26 |
| histone H4 | 0 | 58 | 58 | 0 | 100 | 4 | 14.5 |
| syntaxin binding protein (SBP) | 0 | 54 | 54 | 0 | 100 | 21 | 2.57 |
| histone H2A | 12 | 33 | 45 | 26.67 | 73.33 | 6 | 7.5 |
| kinesin | 35 | 6 | 41 | 85.37 | 14.63 | 41 | 1 |
| serine/threonine protein kinase | 23 | 17 | 40 | 57.50 | 42.50 | 27 | 1.48 |
| beta galactofuranosyl glycosyltransferase | 5 | 35 | 40 | 12.50 | 87.50 | 36 | 1.11 |
| glutamamyl carboxypeptidase | 2 | 34 | 36 | 5.56 | 94.44 | 4 | 9 |
| casein kinase | 0 | 35 | 35 | 0 | 100 | 9 | 3.89 |
| mitochondrial carrier protein | 33 | 1 | 34 | 97.06 | 2.94 | 33 | 1.04 |
| ATP-dependent DEAD/H RNA helicase | 21 | 10 | 31 | 67.74 | 32.26 | 31 | 1 |
| ***Endonuclease-reverse transcriptase/ Reverse transcriptase (RNA-dependent DNA polymerase)*** | 4 | 26 | 30 | 13.33 | 86.67 |  |  |
| ***receptor-type adenylate cyclase*** | 3 | 26 | 29 | 10.34 | 89.66 |  |  |
| ***calpain-like cysteine peptidase*** | 26 | 2 | 28 | 92.86 | 7.14 | 10 | 2.8 |
| cystathionine beta-synthase | 4 | 24 | 28 | 14.29 | 85.71 | 6 | 4.67 |
| N-acetyltransferase complex ARD1 subunit | 1 | 26 | 27 | 3.70 | 96.30 | 23 | 1.17 |
| ***chaperone DNAJ protein*** | 24 | 2 | 26 | 92.31 | 7.69 | 23 | 1.13 |
| amastin | 8 | 18 | 26 | 30.77 | 69.23 | 11 | 2.36 |
| ATP-dependent RNA helicase | 19 | 6 | 25 | 76.00 | 24.00 | 24 | 1.04 |
| ***protein associated with differentiation 4*** | 5 | 18 | 23 | 21.74 | 78.26 |  |  |
| ***reverse transcriptase (RNA-dependent DNA polymerase)*** | 1 | 22 | 23 | 4.35 | 95.65 |  |  |
| ***leucine-rich repeat protein (LRRP)*** | 14 | 7 | 21 | 66.67 | 33.33 |  |  |
| ***amino acid transporter*** | 4 | 17 | 21 | 19.05 | 80.95 |  |  |
| serine/threonine protein phosphatase | 15 | 5 | 20 | 75.00 | 25.00 | 16 | 1.25 |
| ABC transporter | 13 | 6 | 19 | 68.42 | 31.58 | 16 | 1.19 |
| ***cation transporter*** | 2 | 16 | 18 | 11.11 | 88.89 |  |  |
| ***cysteine peptidase*** | 1 | 17 | 18 | 5.56 | 94.44 |  |  |
| ***tryptophanyl-tRNA synthetase*** | 1 | 17 | 18 | 5.56 | 94.44 |  |  |
| dynein light chain | 9 | 8 | 17 | 52.94 | 47.06 | 15 | 1.13 |
| calmodulin | 8 | 9 | 17 | 47.06 | 52.94 | 12 | 1.42 |
| elongation factor 1-alpha | 2 | 15 | 17 | 11.76 | 88.24 | 5 | 3.4 |
| sigma-adaptin 3 | 0 | 17 | 17 | 0 | 100 | 4 | 4.25 |
| tyrosine aminotransferase | 4 | 12 | 16 | 25.00 | 75.00 | 8 | 2 |
| ubiquitin-conjugating enzyme E2 | 15 | 0 | 15 | 1000 | 0 | 14 | 1.07 |
| ***DNA repair protein*** | 10 | 5 | 15 | 66.67 | 33.33 |  |  |
| ***hexose transporter*** | 13 | 1 | 14 | 92.86 | 7.14 |  |  |
| cyclophilin | 12 | 2 | 14 | 85.71 | 14.29 | 6 | 2.33 |
| ***GTP-binding protein*** | 11 | 3 | 14 | 78.57 | 21.43 |  |  |
| mitochondrial RNA binding complex 1 subunit | 10 | 4 | 14 | 71.43 | 28.57 | 13 | 1.08 |
| ***DnaJ chaperone protein*** | 6 | 8 | 14 | 42.86 | 57.14 |  |  |
| ***amino acid permease*** | 13 | 0 | 13 | 100 | 0 |  |  |
| ***chaperone protein DNAj*** | 11 | 2 | 13 | 84.62 | 15.38 |  |  |
| ***ubiquitin hydrolase*** | 10 | 3 | 13 | 76.92 | 23.08 |  |  |
| expression site-associated gene (ESAG-like) protein | 6 | 7 | 13 | 46.15 | 53.85 | 11 | 1.18 |
| Lys-63-specific deubiquitinase BRCC36 | 1 | 12 | 13 | 7.69 | 92.31 | 3 | 4.33 |
| ***ubiquitin carboxyl-terminal hydrolase*** | 12 | 0 | 12 | 100 | 0 |  |  |
| ***heat shock protein 70 (hsp70)*** | 2 | 10 | 12 | 16.67 | 83.33 |  |  |
| folate/pteridine transporter | 1 | 11 | 12 | 8.33 | 91.67 | 4 | 3 |
| ***Histone H2B variant V*** | 1 | 11 | 12 | 8.33 | 91.67 | 3 | 4 |
| flagellar calcium-binding 24 kDa protein | 0 | 12 | 12 | 0 | 100 | 1 | 12 |
| ***oligosaccharyl transferase subunit*** | 0 | 12 | 12 | 0 | 100 |  |  |
| serine/threonine-protein phosphatase PP1 | 8 | 3 | 11 | 72.73 | 27.27 | 6 | 1.83 |
| ***ADP-ribosylation factor*** | 7 | 4 | 11 | 63.64 | 36.36 |  |  |
| chaperonin HSP60, mitochondrial precursor | 5 | 6 | 11 | 45.45 | 54.55 | 6 | 1.83 |
| ***lipase*** | 5 | 6 | 11 | 45.45 | 54.55 |  |  |
| glycerate kinase | 0 | 11 | 11 | 0 | 100 | 1 | 11 |
| * TCFPs marked in bold italics were not considered for further analysis because of their low precision functional annotation.  **Total count/contig. | | | | | | | |

**Supplementary Table 5. Compartmental distribution and features of TS-GI sequences.**

| **ID** | **Product** | **Length** | **Region** | **Motif** | **TS activity*** | **Repeat** |
| --- | --- | --- | --- | --- | --- | --- |
| RA_10_3263_1 | TS-GI | 706 | Core | NAAYS | Yes |  |
| RA_133_848_1 | TS-GI | 828 | Disruptive |  | ? | KGKATGSSA |
| RA_178_263_1 | TS-GI | 717 | Core | NSAYS | Yes |  |
| RA_20_635_2 | TS-GI | 782 | Core |  | ? |  |
| RA_21_1012_1 | TS-GI | 876 | Disruptive |  | ? | KGKATGSSA |
| RA_21_1044_1 | TS-GI | 852 | Disruptive |  | ? | KGKATGSSA |
| RA_212_36_1 | TS-GI | 804 | Disruptive |  | ? | KGKATGSSA |
| RA_327_356_2 | TS-GI | 782 | Core |  | ? |  |
| RA_48_1180_1 | TS-GI | 718 | Core | NSAYS | Yes |  |
| RA_48_826_1 | TS-GI | 718 | Core | NSAYS | Yes |  |
| RA_574_38_1 | TS-GI | 691 | N/A | NAAYS | Yes |  |
| RA_6_3867_1 | TS-GI | 892 | Disruptive | NSAYS | Yes | SAPA** |
| RA_6_3139_2 | TS-GI | 1178 | Disruptive | NAAYS | Yes | SAPA** |
| RA_6_2988_3 | TS-GI | 926 | Disruptive | NSAYS | Yes | SAPA** |
| RA_71_1119_2 | TS-GI | 812 | Disruptive | NSAYS | Yes | SAPA** |
| RA_82_1037_2 | TS-GI | 824 | Disruptive | NSAYS | Yes | SAPA** |
| RA_82_355_2 | TS-GI | 728 | Disruptive | NSAYS | Yes |  |
| N/A: This contig is out of the resolution limit of GCanner | | | | | | |
| * TS activity was inferred upon similarity to the reference molecule [^54^](https://www.zotero.org/google-docs/?DrukKI). | | | | | | |
| ** SAPA repeat: DSSAHSTPSTPV | | | | | | |

**Supplementary Table 6. Coordinates and feature densities of the RA-mapped regions analysed in this study.**

See the Supplementary_table_6.csv file in https://github.com/BuscagliaLab/RA-genome

**Supplementary Table 7. Count of regions of each type showing the indicated feature.**

| **Feature** | **Core genome** | **Disruptive genome*** | | | |
| --- | --- | --- | --- | --- | --- |
|  |  | **TcMUC/ MASP/ TS-GV (*n*=100)** | **RHS/ DGF1/ TS-GII (*n*=176)** | **GP63 (*n*=32)** | **Other (*n*=146)** |
| TCHP | 389 | 83 | 111 | 27 | 132 |
| L1Tc | 17 | 46 | 31 | 12 | 10 |
| RNase H | 16 | 38 | 15 | 11 | 4 |
| NARTc | 24 | 18 | 22 | 1 | 6 |
| VIPER | 50 | 30 | 75 | 4 | 21 |
| SIRE | 214 | 65 | 121 | 24 | 54 |
| C/D snoRNA | 13 | 34 | 79 | 6 | 13 |
| H/ACA snoRNA | 14 | 6 | 2 | 6 | 11 |
| TS-GI | 7 | 2 | 3 | 1 | 3 |
| TOR kinase 1 | 18 | 18 | 29 | 2 | 10 |
| GT | 3 | 1 | 14 | 4 | 12 |
| glutamamyl carboxypeptidase | 2 | 0 | 0 | 1 | 2 |
| amastin | 5 | 0 | 0 | 2 | 2 |
| ESAG-like protein | 2 | 0 | 5 | 1 | 0 |
| TS-GIII | 3 | 0 | 8 | 0 | 2 |
| TS-GVII | 2 | 13 | 28 | 0 | 2 |
| TS-GVIII | 4 | 0 | 36 | 0 | 3 |
| TcSMUGL | 0 | 0 | 0 | 0 | 3 |
| TcSMUGS | 0 | 0 | 0 | 0 | 4 |
| TASV | 0 | 2 | 3 | 0 | 5 |
| TSSA | 0 | 0 | 0 | 0 | 1 |
| histone H4 | 0 | 0 | 0 | 0 | 4 |
| histone H2A | 1 | 0 | 0 | 0 | 2 |
| casein kinase | 0 | 1 | 0 | 0 | 1 |
| cystathionine beta-synthase | 4 | 0 | 0 | 0 | 2 |
| dynein light chain | 6 | 0 | 0 | 0 | 2 |
| calmodulin | 7 | 1 | 0 | 0 | 3 |
| elongation factor 1-alpha | 2 | 0 | 0 | 0 | 3 |
| sigma-adaptin 3 | 0 | 0 | 0 | 0 | 3 |
| tyrosine aminotransferase | 4 | 1 | 0 | 0 | 1 |
| folate/pteridine transporter | 1 | 0 | 0 | 0 | 3 |
| chaperonin HSP60, mitochondrial precursor | 5 | 0 | 0 | 0 | 1 |
| glycerate kinase | 0 | 0 | 0 | 0 | 1 |
| TS-GIV | 0 | 4 | 23 | 0 | 0 |
| TS-GVI | 0 | 30 | 6 | 0 | 0 |
| beta galactofuranosyl glycosyltransferase | 5 | 5 | 25 | 0 | 0 |
| N-acetyltransferase complex ARD1 subunit | 1 | 1 | 16 | 0 | 0 |
| syntaxin binding protein (SBP) | 0 | 20 | 0 | 0 | 0 |
| Lys-63-specific deubiquitinase BRCC36 | 1 | 4 | 0 | 0 | 0 |
| flagellar calcium-binding 24 kDa protein | 0 | 1 | 0 | 0 | 0 |
| *Categories within the disruptive genome were defined by multigene family densities (see text).  GT: UDP-Gal or UDP-GlcNAc-dependent glycosyltransferase | | | | | |

**
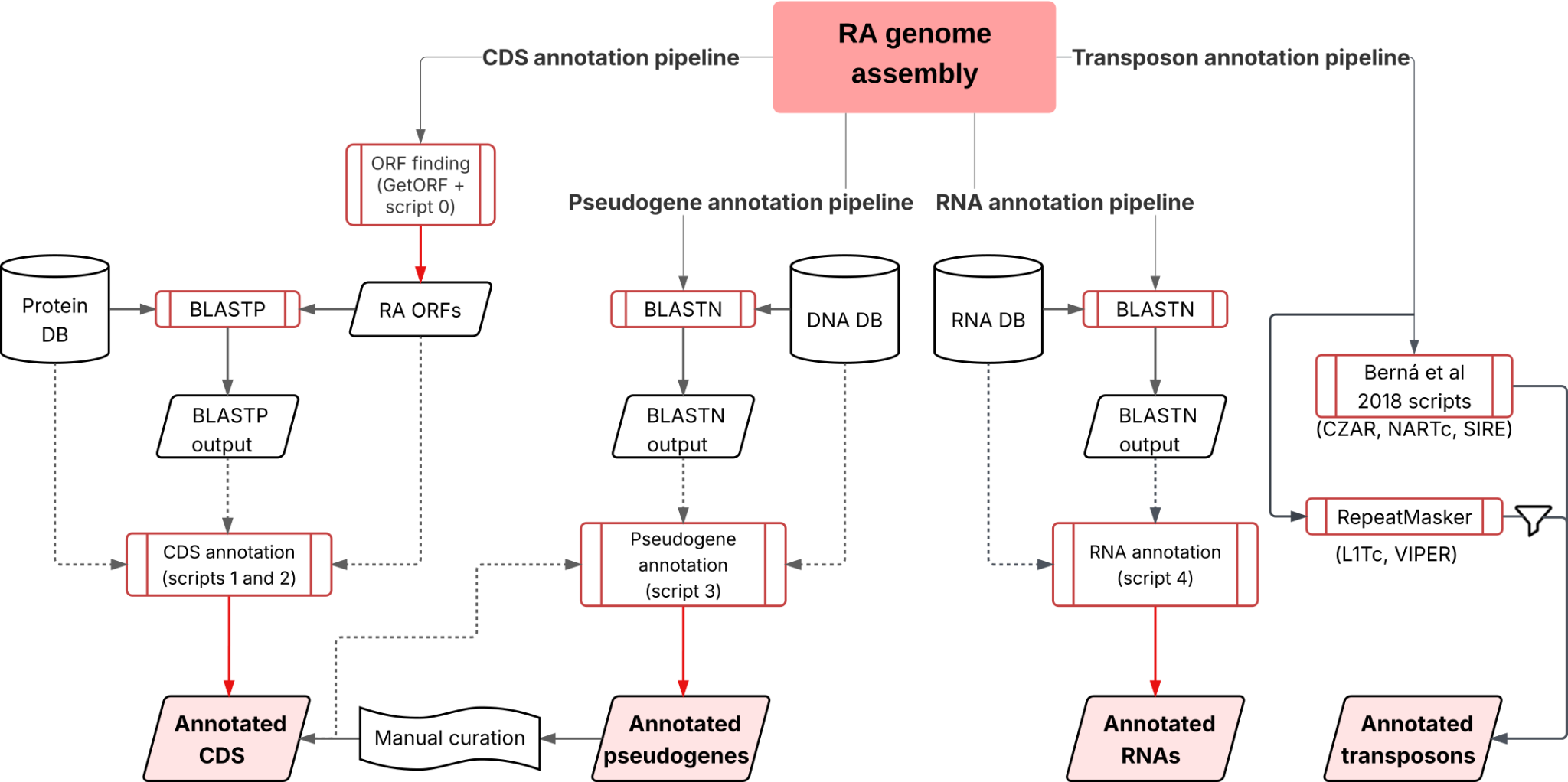
**

**Supplementary Figure 1.** Schematic workflow for RA genome annotation.

**
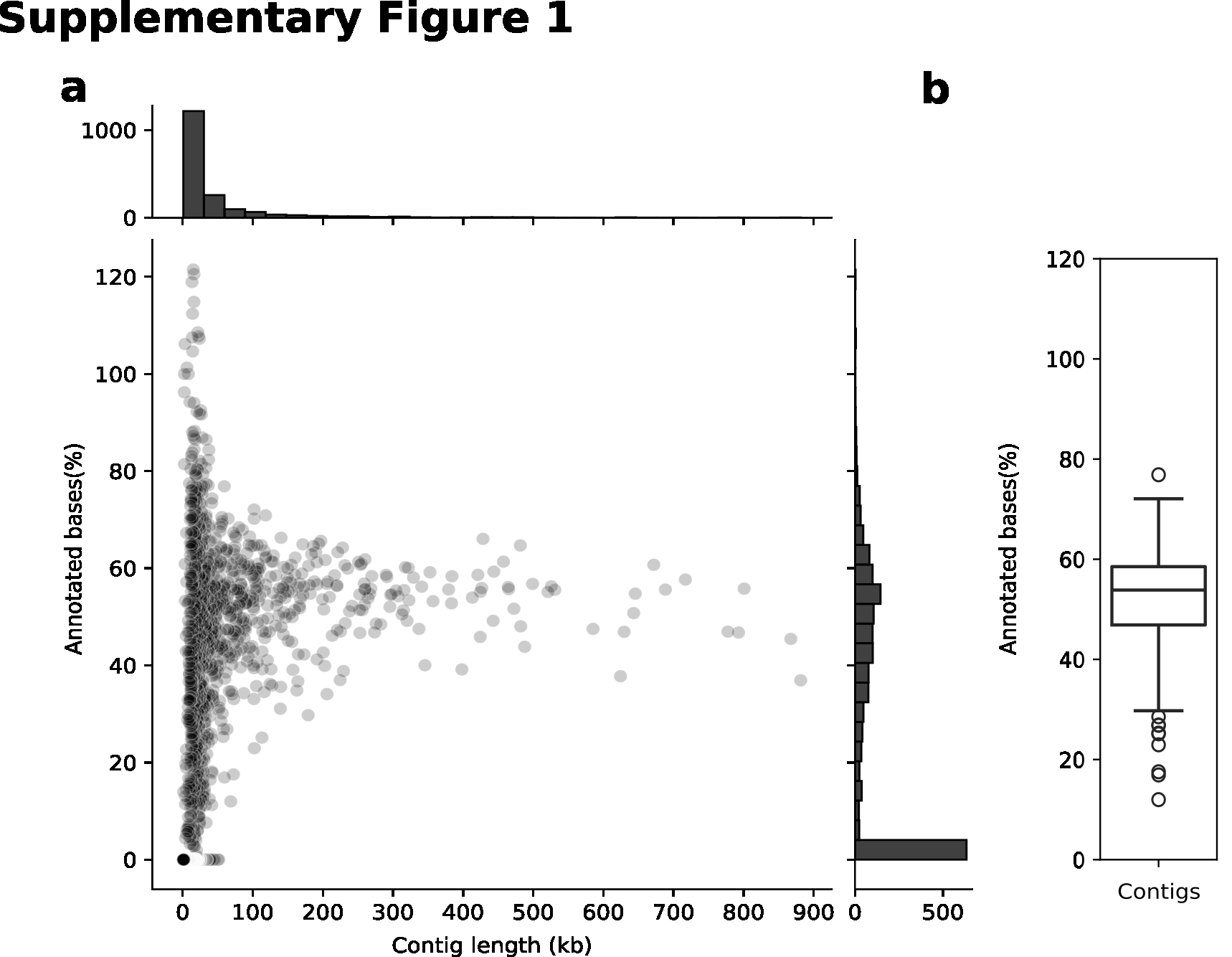
**

**Supplementary Figure 2**. **Distribution of annotated features on RA genomic contigs**. **a.** Joint plot displaying the percentage of annotated bases (calculated as the sum of all annotated bases on a contig divided by the contig length, multiplied by 100) plotted against contig length. Each dot represents an individual contig. **b**. Box plot illustrating the percentage of annotated bases, limited to contigs over 50 Kb displaying at least one annotated feature (*n* = 384). The box represents the first quartile, median, and third quartile, with whiskers extending 1.5 times the interquartile range (IQR).


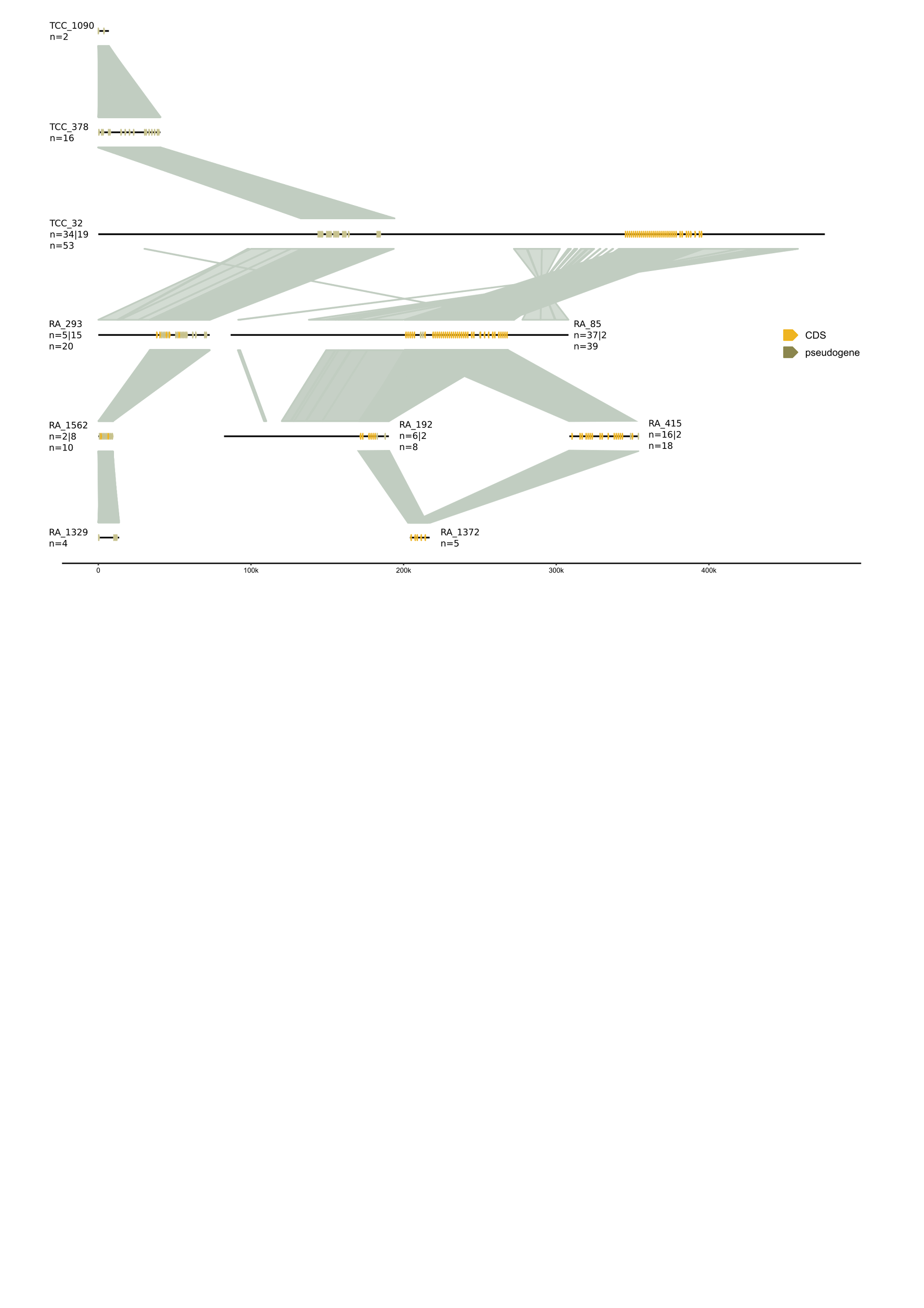


**Supplementary Figure 3**. **TcSMUGL synteny**. Synteny scheme of the contigs with TcSMUGL-annotated genes (yellow arrows) and pseudogenes (green arrows) in TCC and RA strains. Each contig is represented by an in scale horizontal black line, and the corresponding number of TcSMUGL CDS | pseudogenes (above), and total TcSMUGL annotations (below) are shown on the side.


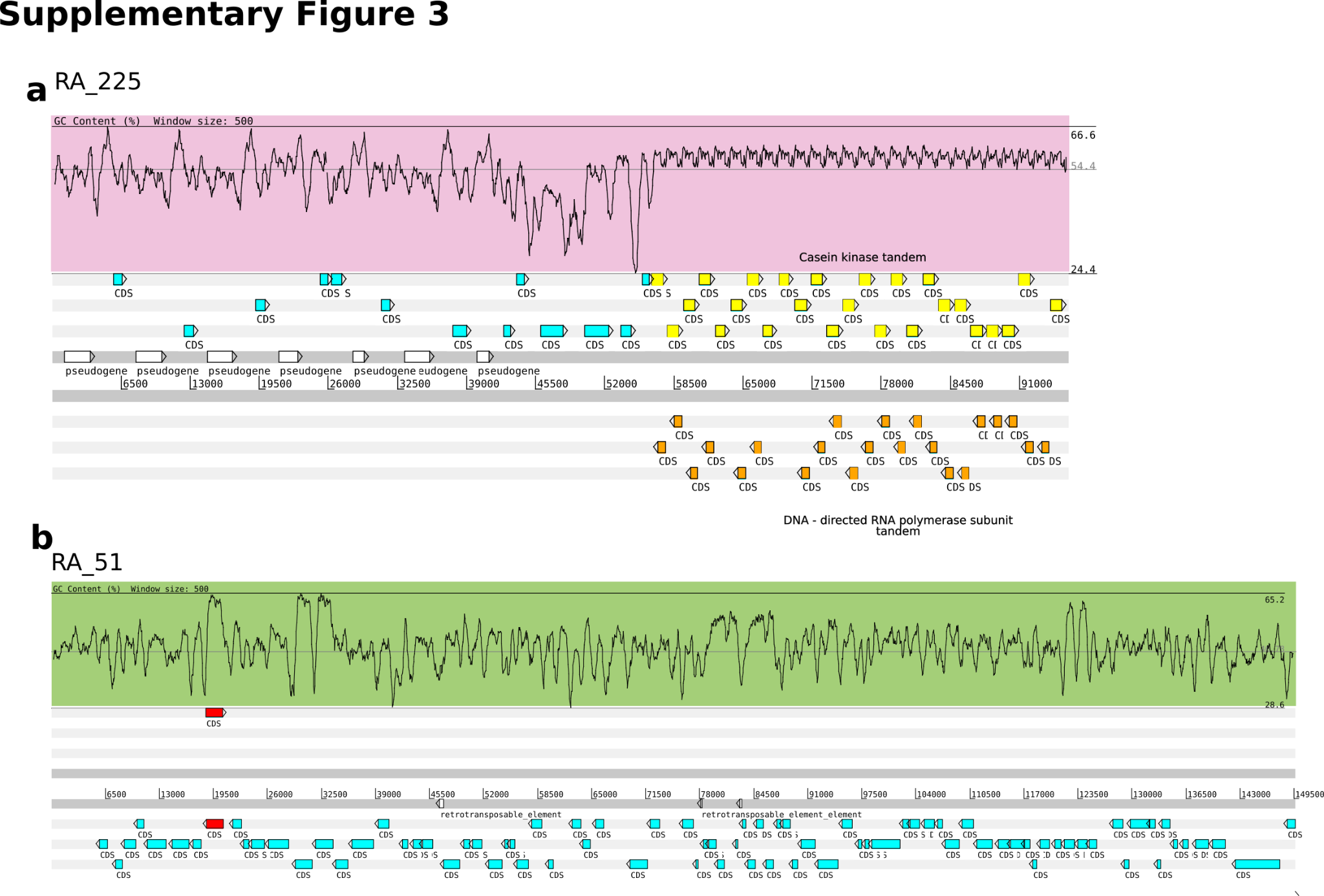


**Supplementary Figure 4**. **CDS overlap in the RA genome.** **a.** Overlap between casein kinase proteins and the DNA-directed RNA polymerase subunit. The Artemis view of contig RA_225 displays GC content (top) and annotated features across all six reading frames (bottom). Casein kinase proteins are highlighted in yellow, DNA-directed RNA polymerase subunits in orange, and other genes and features in light blue and white boxes. InterProScan analysis indicated that casein kinases belong to the Ser/Thr kinase family (ID: IPR050235) and contain protein kinase domains (ID: IPR000719). A similar analysis of the DNA-directed RNA polymerase subunit did not reveal any predicted functional domains. An overlap between CDS from the same families was evidenced for contig RA_36 (not shown) **b.** Overlap between Heat-shock proteins 70 (HSP70) CDS. The Artemis view of contig RA_51 shows GC content (top) and annotated features across all six reading frames (bottom). HSP70 are marked in red, while other genes and features are displayed in light blue and white boxes. Domain searches showed that the upper HSP70 belongs to a glutamate dehydrogenase NAD-specific family (ID: IPR019651). The lower HSP70 was validated as a member of the heat-shock protein family (ID: IPR013126).

**
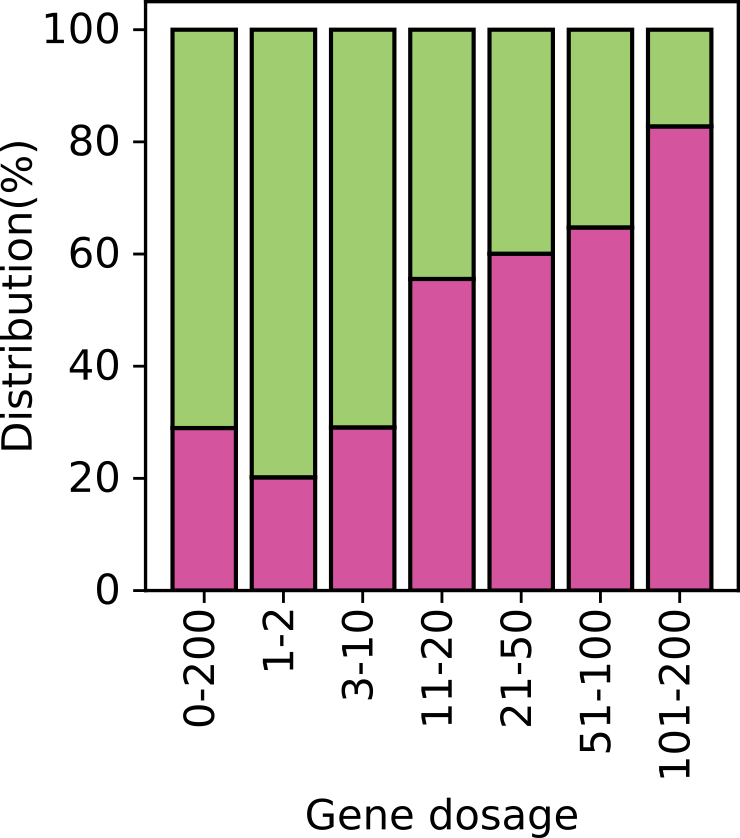
**

**Supplementary Figure 5**. **Dosage and compartment distribution of TCFP sequences.** Each bar corresponds to the dosage (copy number of sequences sharing the same annotation) range of TCFP indicated at the bottom. For each range, the distribution of copies among core and disruptive compartments was calculated and expressed as percentage.
